# Virtual Reality Sensing Technologies in Student Soccer Training: A Systematic Review of Tracking Systems and Perceptual-Cognitive, Physical, Tactical, and Educational Outcomes

**DOI:** 10.3390/s26175381

**Published:** 2026-08-26

**Authors:** Jaejun Park, Zainab Ghazanfar, Saba Ghazanfar Ali, Sang Wan Jeon, Younhyun Jung

**Affiliations:** 1 School of Computing, Gachon University, Seongnam-si 13120, Republic of Korea; 3636qq@gachon.ac.kr (J.P.); zainab@gachon.ac.kr (Z.G.); 2 Department of Computer Science and Engineering, Shanghai Jiao Tong University, Shanghai 200240, China; 3 Education Department, Mokpo National University, Muan-gun 58554, Republic of Korea

**Keywords:** virtual reality, sensing technologies, head-mounted display, tracking systems, wearable sensors, perceptual-cognitive training, student soccer, systematic review

## Abstract

**Highlights:**

**What are the main findings?**

- Six-degrees-of-freedom HMD systems and 360° projection platforms have been used to examine neuromuscular control, scanning behavior, and tactical decision-making in student soccer; however, comparative evidence between hardware configurations is lacking.
- EEG- and eye-tracking-augmented VR systems enable objective measurement of anticipatory neural preparation and gaze efficiency in youth soccer players.
- Sensor hardware heterogeneity—in latency, tracking fidelity, and degrees of freedom—limits cross-study comparability across the 20 included studies.

**What are the implications of the main findings?**

- VR sensing systems may serve as complementary performance-assessment and instructional tools in school, academy, and university soccer settings.
- Standardized sensor reporting, longitudinal transfer studies, feasibility evaluation, and implementation research are needed before large-scale adoption of VR in student soccer education can be recommended.

**Abstract:**

Soccer requires rapid tactical decision-making, perceptual-cognitive processing, and coordinated physical execution, making it a relevant application domain for virtual reality (VR). VR systems integrate head-mounted displays, inertial measurement units, eye-tracking sensors, and electroencephalographic interfaces to support training, assessment, rehabilitation, and engagement. However, evidence remains fragmented across populations, study designs, sensing configurations, and research purposes, limiting conclusions about effectiveness and real-world application. Following PRISMA 2020, this systematic review synthesized 20 empirical studies published between January 2020 and 16 August 2026 involving school, academy, collegiate, and selected indirect adult soccer populations. Studies were classified by sensor modality, tracking configuration, evidence purpose, and outcome domain. Controlled intervention studies reported possible short-term improvements in selected sensorimotor, tactical, perceptual-cognitive, technical, and motivational outcomes. In contrast, cross-sectional and profiling studies showed that some VR tasks distinguished players by expertise, age, or competitive level; these findings support assessment or discriminative validity but do not demonstrate that VR training improves performance. Rehabilitation and injury-related evidence was limited and partly derived from adult or clinical populations, whereas engagement studies suggested potential benefits for motivation, attention, and participation. Six-degrees-of-freedom HMDs, 360° projection systems, eye tracking, and EEG generated different types of evidence, but no study directly compared hardware configurations within the same sample. The main contribution of this review is an integrated evidence-purpose and sensor-based synthesis that distinguishes training effectiveness from assessment validity across student-soccer applications. Overall, evidence remains promising but preliminary because of small and mixed populations, methodological heterogeneity, incomplete technical reporting, possible publication and language bias, short follow-up, and limited transfer to full-match performance. Standardized sensor reporting, controlled interventions, and longitudinal transfer assessments are required. Protocol registration: OSF.

## 1. Introduction

**Figure 1:**
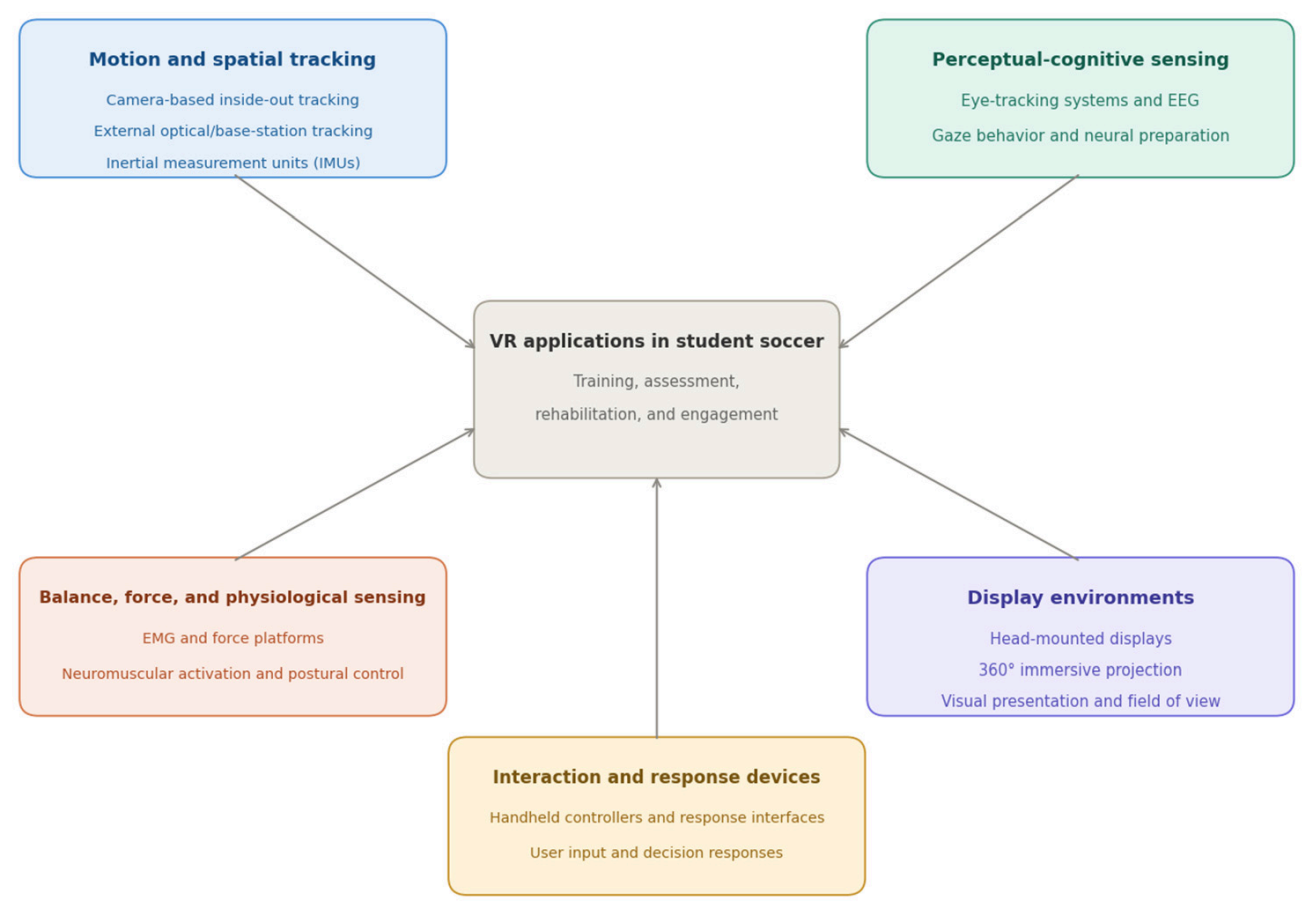
Classification of display environments, tracking technologies, and auxiliary sensing modalities identified across the studies on VR applications in student and youth soccer.

Virtual reality (VR) has evolved from a niche visualization tool into established immersive training, assessment, and interaction technology. Its operation depends on integrated sensing systems that capture, interpret, and respond to users’ movements and task-related actions in real time [1,2]. In sports-training contexts, VR sensing architecture typically combines three layers. The first layer comprises motion and spatial-tracking technologies, including camera-based inside-out tracking, external optical base-station tracking, and inertial measurement units (IMUs), which capture head, body, and controller movement. The second layer comprises perceptual-cognitive sensing and monitoring technologies, including eye-tracking systems and electroencephalographic (EEG) interfaces, which measure gaze behavior, visual attention, and neural preparation. The third layer comprises balance, force, and physiological sensing technologies, including electromyographic (EMG) electrodes and force platforms, which quantify neuromuscular activation, postural control, and movement stability. In sports-training contexts, VR sensing architectures may incorporate motion and spatial tracking, eye tracking, neurophysiological sensing, and other peripheral sensing technologies depending on the application [3,4,5,6,7]. However, the included studies differed substantially in how these sensing components were configured and reported, and many did not provide complete technical details regarding tracking architecture, latency, frame rate, calibration, or sampling rate. Consequently, the scientific evidence on how specific sensing configurations influence outcomes in student and youth soccer remains fragmented and difficult to compare. Figure 1 illustrates the classification of sensing technologies across the VR systems identified in the included studies.

Among sports, soccer is an ideal testbed for VR-based interventions because it is highly dynamic and team-oriented, demanding continuous integration of perceptual-cognitive, tactical, and physical skills. Players must rapidly process spatial information, coordinate with teammates, and make high-stakes tactical decisions in real time [1,2]. These demands make soccer-specific VR systems a natural application for the sensing layers described above: optical and IMU-based motion tracking captures positioning and movement, while eye-tracking and EEG sensors quantify the scanning and anticipation behaviors central to tactical performance. Additionally, soccer has a substantial injury burden in youth populations, with lower-extremity injuries representing a major component of this burden [8], making VR a potentially relevant platform for controlled neuromuscular rehearsal and injury-risk-related training. However, whether such applications reduce injury incidence or improve return-to-play outcomes remains uncertain.

Traditional training in student soccer typically relies on on-field drills, small-sided games, whiteboard, video tactical sessions, and coach-led feedback. While these approaches develop core physical and tactical competencies, they have notable constraints. Repeated high-intensity and contact-based soccer exposure is associated with relevant injury risk in youth populations [8]. Tactical instruction delivered through static means (e.g., video replay, chalk talk) often lacks perceptual realism and does not fully recreate the visual complexity or decision speed of match play. Moreover, in many schools, academies, and university settings, access to individualized feedback, consistent supervision, and scenario-specific repetition is limited. As a result, the development of perceptual-cognitive skills such as scanning, anticipation, and situational awareness can be inconsistent across players and contexts [1,2].

In parallel with these constraints, VR has emerged in sports education as an increasingly adopted training technology [9,10]. Systematic reviews in adjacent sports domains, including tennis and exergaming, have reported potential benefits for skill development, transfer of learning, and engagement in student and youth athlete populations [10]. VR can create immersive and repeatable practice environments that may reduce exposure to selected physical demands; however, the safety advantages of these environments have not been established across student-soccer populations.

By modulating task constraints, feedback mechanisms, and environmental cues, VR supports targeted motor and cognitive training and can accelerate both physical execution and situational decision-making abilities [3,11]. In soccer specifically, VR has been applied to support tactical perception, such as reading play under pressure, spatial awareness, passing decisions, and anticipation of opponents’ and teammates’ movement trajectories [1,2]. It has also been used to expose players to selected match-like demands without a full physical load, suggesting a possible role in neuromuscular retraining and return-to-play-related rehearsal. These applications remain provisional because injury reduction and return-to-play effectiveness have not been directly established [1,12].

Beyond direct performance outcomes, VR also has documented educational applications for student populations across schools, academies, and university levels. These athletes are in critical developmental periods during which motor coordination, attention regulation, working memory, and perceptual-cognitive expertise are still being formed. Unlike elite professionals, student athletes often face inconsistent training exposure and variable motivation. Some studies suggest that VR-integrated training may increase engagement, enjoyment, and self-directed practice under specific educational conditions. VR may function as both a performance-support and pedagogical tool, although its educational effectiveness and scalability require further evaluation [10,13].

Given the multidimensional nature of soccer, this review evaluates VR interventions across five interrelated domains essential to student-athlete development: (i) physical training (balance, agility, motor coordination, and movement efficiency) [14,15,16]; (ii) tactical understanding (spatial awareness, anticipation, and decision-making) [4,6,13]; (iii) injury-related and rehabilitation outcomes (neuromuscular control, postural stability, controlled movement rehearsal, and injury-risk surrogate measures) [14,15,16]; (iv) engagement (motivation, enjoyment, and training adherence) [13]; and (v) cognitive development (attention, working memory, and perceptual-cognitive expertise) [4,5,6]. Together, these domains illustrate how VR has the potential to contribute not only to improved athletic performance but also to broader developmental and educational outcomes for student soccer players. Therefore, this review aims to systematically evaluate the impact of VR interventions on physical, tactical, cognitive, motivational, and injury-related outcomes in student soccer populations between January 2020 and August 2026.

Despite the growing application of VR in sports science and education, the evidence base remains scattered and domain-specific. Many studies report benefits for perceptual-cognitive training [2,9] and motor learning, although few synthesize these outcomes specifically within student soccer. Domain-specific reviews have begun to address individual sports—for example, VR in tennis has been examined for its effects on performance, engagement, and skill transfer [10]—yet soccer remains without a dedicated synthesis of VR sensing technologies and their developmental outcomes. Moreover, most existing reviews emphasize general exercise, clinical rehabilitation [11], or elite performance, and therefore overlook the distinct developmental, cognitive, and motivational needs of youth and student soccer. This creates a gap for educators, coaches, and curriculum designers who want to understand whether VR meaningfully helps student learning, decision-making, and safe performance in soccer environments such as schools, academies, and universities.

Specifically, the review investigates: (1) which VR technologies, training formats, and task designs have been used in student soccer since 2020, and in which countries and years; (2) whether these interventions demonstrate effectiveness across the five developmental domains, including outcomes such as training effects, on-field transfer, feasibility, safety, and implementation burden; (3) how VR compares to alternative or control conditions (e.g., routine drills, desktop simulations), including effect sizes and study designs; and (4) who the participants were—age, sample size, and context (school, academy, university)—along with any reporting limitations or risks of bias.

Although selected studies reported usability-related outcomes such as perceived effort, hedonic motivation, immersion ratings, participation, and user experience, the available literature did not provide sufficiently consistent evidence for a comprehensive HCI synthesis. Constructs such as presence, embodiment, cognitive workload, learnability, accessibility, interaction quality, cybersickness, and standardized usability were rarely or inconsistently assessed. Accordingly, these outcomes were treated as secondary engagement, usability, or feasibility findings rather than as a complete HCI evaluation framework. The remainder of this paper is organized as follows: Section 2 describes the materials and methods; Section 3 presents findings across the five domains; Section 4 discusses implications, compares prior reviews, and outlines future directions; and Section 5 presents the conclusion.

## 2. Materials and Methods

### 2.1. Study Design

This review followed the Preferred Reporting Items for Systematic Reviews and Meta-Analyses (PRISMA 2020) guidelines [17] to ensure transparency and methodological rigor in the identification, screening, eligibility assessment, and inclusion of studies. In addition to standard PRISMA data extraction, each included study was assessed for its VR hardware configuration and associated sensing modalities. Studies were classified according to: (i) display type (head-mounted display (HMD) with six-degrees-of-freedom (6DoF) tracking, 360° panoramic projection system, or desktop VR); (ii) motion-tracking sensor modality (optical marker-based, inertial measurement unit (IMU)-based, or electromagnetic); (iii) auxiliary sensing peripherals (eye-tracking cameras, electromyographic (EMG) electrodes, electroencephalographic (EEG) headsets, force platforms, or foot-tracking controllers); and (iv) reported technical parameters where available (motion-to-photon latency, frame rate, degrees of freedom, and field of view). This hardware classification was used to evaluate whether sensor specifications were associated with outcome quality and cross-study comparability. As this study is a systematic review, no physical experimental equipment, materials, or sensing devices were directly used by the authors. Information on VR hardware, tracking systems, interaction devices, and auxiliary sensors was extracted from the included studies. Manufacturer, model, and technical information were recorded only when explicitly reported in the original publications; unreported specifications were not inferred. A consistent coding framework was applied, encompassing country, participants, soccer context, study design, VR intervention type, display and tracking configuration, interaction device, control condition, outcome measures, and main findings. Usability, motivation, perceived effort, immersion, participation, feasibility, and user-experience outcomes were extracted when explicitly reported. Because the included studies did not consistently assess broader HCI constructs such as presence, embodiment, accessibility, cognitive workload, learnability, or cybersickness, no comprehensive HCI quality score or formal HCI synthesis was undertaken. This review was not prospectively registered before study identification, screening, or data extraction. To improve transparency, the completed protocol was registered retrospectively on the Open Science Framework (OSF): https://doi.org/10.17605/OSF.IO/WX2G5 accessed on 30 June 2026. Because registration occurred after the review process had begun, it could not prevent possible data-informed changes to eligibility, classification, or synthesis decisions. This limitation is discussed further in Section 4.3.

### 2.2. Information Sources, Search Strategy, and Record Management

A systematic search was conducted across five electronic databases: PubMed/MEDLINE, Scopus, Web of Science Core Collection, SPORTDiscus, and ERIC. Studies published between 1 January 2020 and 16 August 2026 were considered eligible.

The reproducibility/update search conducted on 16 August 2026 retrieved 42 records from PubMed/MEDLINE, 96 from Scopus, 71 from Web of Science Core Collection, 34 from SPORTDiscus, and 15 from ERIC. These counts represent the records retrieved during the August 2026 rerun and should not be interpreted as reconstructions of the unavailable database-specific counts from the original historical search. Complete database-specific search syntax, field codes, applied restrictions, exact execution date, and retrieval counts are provided in Supplementary Table S1. All retrieved records were exported to EndNote 2025 (Clarivate, Philadelphia, PA, USA), compared with the original reference library, and subjected to duplicate checking before screening. Records not previously assessed were screened using the same predefined eligibility criteria.

The search strategy combined four main concepts: virtual reality, soccer, student or youth populations, and relevant training or developmental outcomes. The core Boolean structure was as follows:

(“virtual reality” OR “immersive virtual reality” OR “immersive VR” OR “360° video”) AND (“soccer” OR “football”) AND (“student” OR “youth” OR “adolescent” OR “academy” OR “school” OR “university” OR “collegiate”) AND (“physical training” OR “skill acquisition” OR “tactical understanding” OR “decision-making” OR “injury prevention” OR “rehabilitation” OR “motivation” OR “engagement” OR “cognitive” OR “perceptual-cognitive”).

This core strategy was adapted to the syntax and search fields of each database, including Medical Subject Headings and Title/Abstract fields in PubMed, TITLE-ABS-KEY fields in Scopus, Topic fields in Web of Science, and All Text fields in SPORTDiscus and ERIC. No methodological study-design filter was applied to preserve the breadth of potentially relevant studies. English-language and publication-date restrictions were applied.

The original searches identified 230 records in total across the five databases. However, the exact execution dates of the original searches and the individual number of records retrieved from each database were not retained during the original review process and could not be reconstructed reliably. These values were therefore not estimated. The reconstructed database-specific search strategies, field codes, and applied restrictions are reported in Supplementary Table S1.

Records retrieved from the five databases were imported into EndNote 2025 (Clarivate, Philadelphia, PA, USA) and consolidated into a single reference library. Potential duplicate records were identified using EndNote’s duplicate-detection function based on bibliographic fields including title, author, and publication year. Records flagged as possible duplicates were then manually compared, with DOI and journal information consulted where available. Forty-two duplicate records were removed, leaving 188 unique records for title and abstract screening.

The search aimed to identify empirical studies examining VR-based training, assessment, rehabilitation, engagement, or perceptual-cognitive applications in student and youth soccer. Studies were considered relevant when they reported outcomes related to physical training and skill acquisition, tactical understanding and decision-making, injury prevention or rehabilitation, engagement and motivation, or cognitive and perceptual-cognitive development.

### 2.3. Eligibility Criteria

Studies were eligible for inclusion if they met all the following criteria:

In this review, “student and youth soccer” is defined broadly to include (i) school-aged players in physical education or academy structures; (ii) academy or beginner professional athletes in formal development pathways; and (iii) university and collegiate athletes, who remain in organized educational programs and whose training, medical oversight, and return-to-play processes are embedded in institutional sport settings. We use “school,” “academy,” and “university/collegiate” as generic descriptors of organized, institutionally supervised developmental pathways, and not as references to any single country’s education or sport system; equivalent structures in other countries are included under the same definition. No fixed upper age limit was applied to this primary population, because eligibility was defined structurally (enrollment in a school, academy, or university/collegiate program) rather than by a numeric age cutoff; reported participant ages across the directly eligible studies ranged from approximately 11 to 23 years (Table 1; Supplementary Table S3).

- Because the available evidence on VR-based injury-related and rehabilitation applications in student soccer remains limited, we included a small number of studies involving recreational adult players or adult athletes with chronic ankle instability only when the VR intervention targeted soccer-specific skills that are directly relevant to youth safety, developmental training, or return-to-play preparation. These included heading mechanics, neuromuscular control, postural stability, balance under perturbation, and controlled movement rehearsal. These studies were treated as indirect developmental-transfer evidence rather than as direct evidence from student soccer populations. Here, “recreational adult players” refers to adults who play soccer outside of any academy, collegiate, or professional development pathway (i.e., informal, club-level, or non-competitive participation), and who therefore fall outside the structural definition above (mean ages 24.2–28.7 years across the two studies retained, versus 11–23 years for the directly eligible student/youth studies). These two studies were retained solely as indirect, mechanism-level transfer evidence for the injury-related/rehabilitation domain and are not used to draw conclusions about student-soccer training or performance outcomes.
- We did not include studies on fully professional senior squads unless they explicitly compared professionals to academy or novice players to validate VR as an assessment or talent-identification tool for younger cohorts.

**Inclusion Criteria:**

- Intervention: immersive or semi-immersive VR (HMD, 360° projection, or equivalent interactive 3D environment).
- Sport context: soccer-specific tasks (tactical decision-making, perceptual-cognitive tracking, heading, balance, etc.).
- Outcomes: at least one of the five predefined domains (physical training, tactical understanding, injury-related outcomes/rehabilitation, engagement/motivation, cognitive or perceptual-cognitive development).
- Design: empirical quantitative, qualitative, or mixed methods (e.g., randomized controlled trial, quasi-experimental trial, cross-sectional comparison, prospective profiling).
- Language and time frame: English-language studies published between 1 January 2020 and 16 August 2026.

**Exclusion Criteria:**

- Studies in non-soccer sports;
- Studies exclusively on senior elite professionals with no student/academy comparison;
- Simulation/modeling papers without human participants;
- Non-immersive 2D video-only instruction unless it served as an active control arm for an immersive VR condition.

### 2.4. Screening and Study Selection

**Figure 2:**
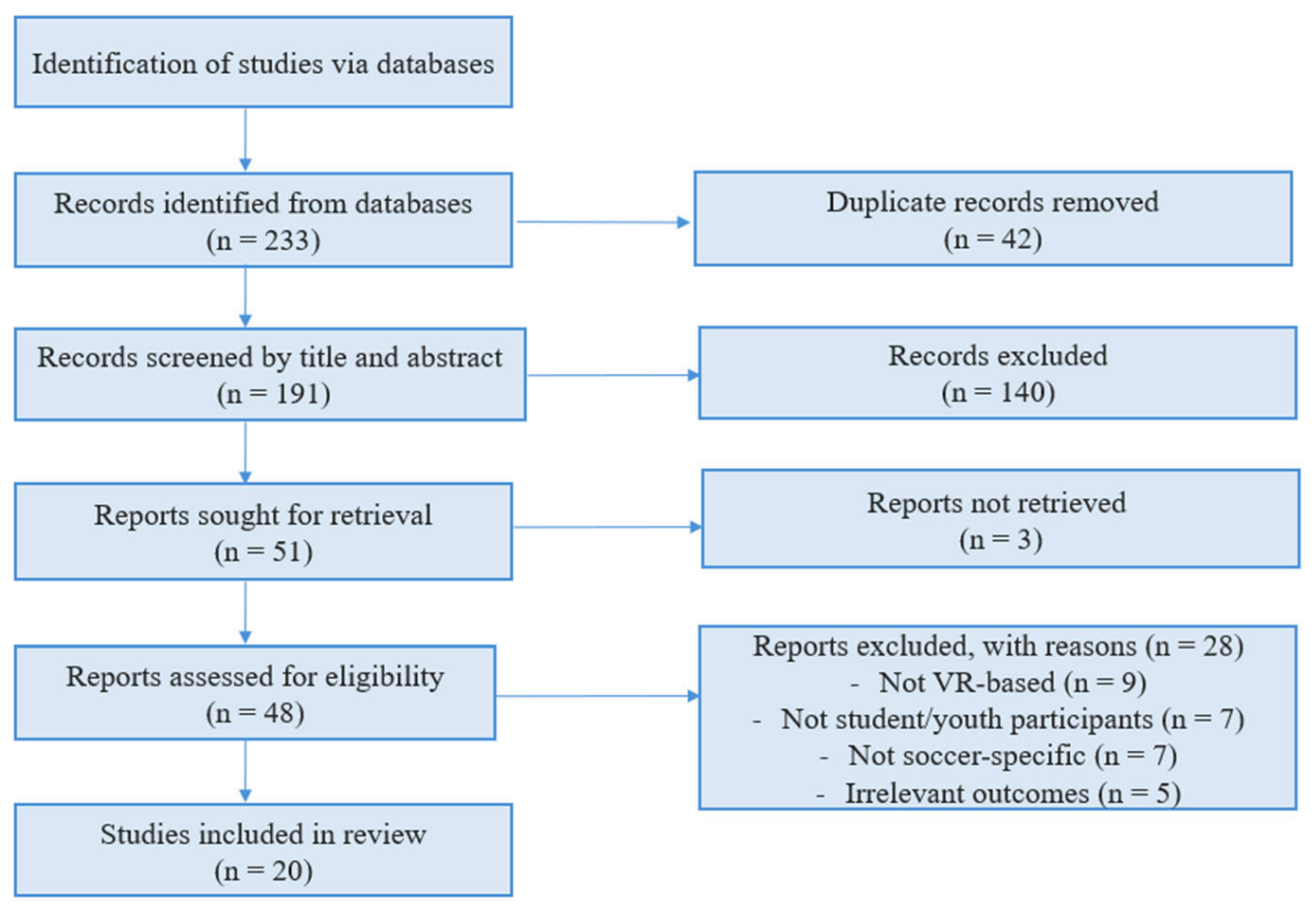
PRISMA flow diagram showing the identification, screening, eligibility assessment, and inclusion of studies on VR interventions in student soccer.

Screening was conducted independently by two reviewers, Zainab Ghazanfar (ZG) and Saba Ghazanfar Ali (SGA), at both the title/abstract and full-text stages, after removing duplicates. Data extraction was performed independently by the same two reviewers using a piloted extraction form. Disagreements at both the screening and extraction stages were resolved through discussion; where consensus could not be reached, adjudication was provided by Prof. Younhyun Jung. Inter-rater agreement was not formally quantified using statistics such as Cohen’s κ; this is noted as a limitation in Section 4.3. Full texts judged potentially eligible were then evaluated in detail. A PRISMA flow diagram (Figure 2) summarizes the identification, screening, eligibility, and inclusion stages.

The original search, covering studies published through 31 March 2026, identified 230 records across the five databases. A reproducibility and update search was conducted on 16 August 2026 using the same database-specific strategies. The rerun retrieved 42 records from PubMed/MEDLINE, 96 from Scopus, 71 from Web of Science Core Collection, 34 from SPORTDiscus, and 15 from ERIC. The retrieved records were exported to EndNote, compared with the original reference library, and deduplicated. Records not previously screened were subsequently assessed using the same predefined eligibility criteria. The update and verification process identified three additional eligible studies, including Makhadmeh and Fayad (2025) [30], which fell within the original review period but had not been captured previously, together with two eligible studies published after the original 31 March 2026 cutoff. Following incorporation of these studies, the final systematic review comprised 20 included studies.

For the revised quantitative synthesis, the original full-text articles were re-examined to extract author-reported group means, standard deviations, percentage changes, test statistics, exact *p*-values, effect estimates, confidence intervals, correlations, odds ratios, and classification-performance measures where available. Values that were not reported and could not be calculated from the published information were explicitly coded as “not reported and not calculable”.

### 2.5. Risk-of-Bias and Methodological-Quality Assessment

The methodological quality and risk of bias of the included studies were evaluated using design-appropriate appraisal tools. Randomized controlled trials were assessed using the Cochrane Risk of Bias 2 (RoB 2) tool [31], covering bias arising from the randomization process, deviations from intended interventions, missing outcome data, outcome measurement, and selection of the reported result. Non-randomized intervention and quasi-experimental studies were assessed using the Risk Of Bias In Non-randomized Studies of Interventions (ROBINS-I) tool [32], covering confounding, participant selection, intervention classification, deviations from intended interventions, missing data, outcome measurement, and selection of reported results. Cross-sectional, comparative, and perceptual-cognitive profiling studies were assessed using the Downs and Black checklist [33], restricted to items applicable to non-randomized and cross-sectional designs. These items addressed reporting quality, external validity, internal validity, confounding, participant selection, outcome measurement, and statistical power. Items that were not applicable to a particular study design were recorded as not applicable and excluded from the denominator.

Two reviewers, Zainab Ghazanfar and Saba Ghazanfar Ali, independently assessed each study. Disagreements were resolved through discussion, with adjudication by Younhyun Jung when consensus could not be reached. For RoB 2 and ROBINS-I, overall judgments were derived according to the respective tool guidance. Downs and Black results were summarized as the number and percentage of applicable criteria satisfied and were categorized as lower, moderate, or greater methodological concern according to the prespecified thresholds reported in Supplementary Tables S2.1–S2.4. Risk-of-bias assessments were used to inform the narrative synthesis but were not used as exclusion criteria. Detailed study-level judgments are reported in Supplementary Tables S2.1–S2.4.

## 3. VR Applications and Evidence Types in Student and Youth Soccer

The included studies differed not only in the outcomes examined but also in their primary research purpose. To avoid conflating training effects with assessment findings, the evidence was classified into four categories: (i) training-effectiveness studies, in which repeated VR exposure was evaluated using pre–post, controlled, or randomized intervention designs; (ii) assessment-validity studies, in which VR tasks were used to distinguish expertise levels, characterize perceptual-cognitive profiles, or predict player development without testing a training effect; (iii) rehabilitation and injury-related studies, which examined neuromuscular control, balance, heading rehearsal, postural stability, or return-to-play-related outcomes; and (iv) engagement, usability, and educational studies, which evaluated motivation, enjoyment, perceived effort, participation, feasibility, or user experience.

**Figure 3:**
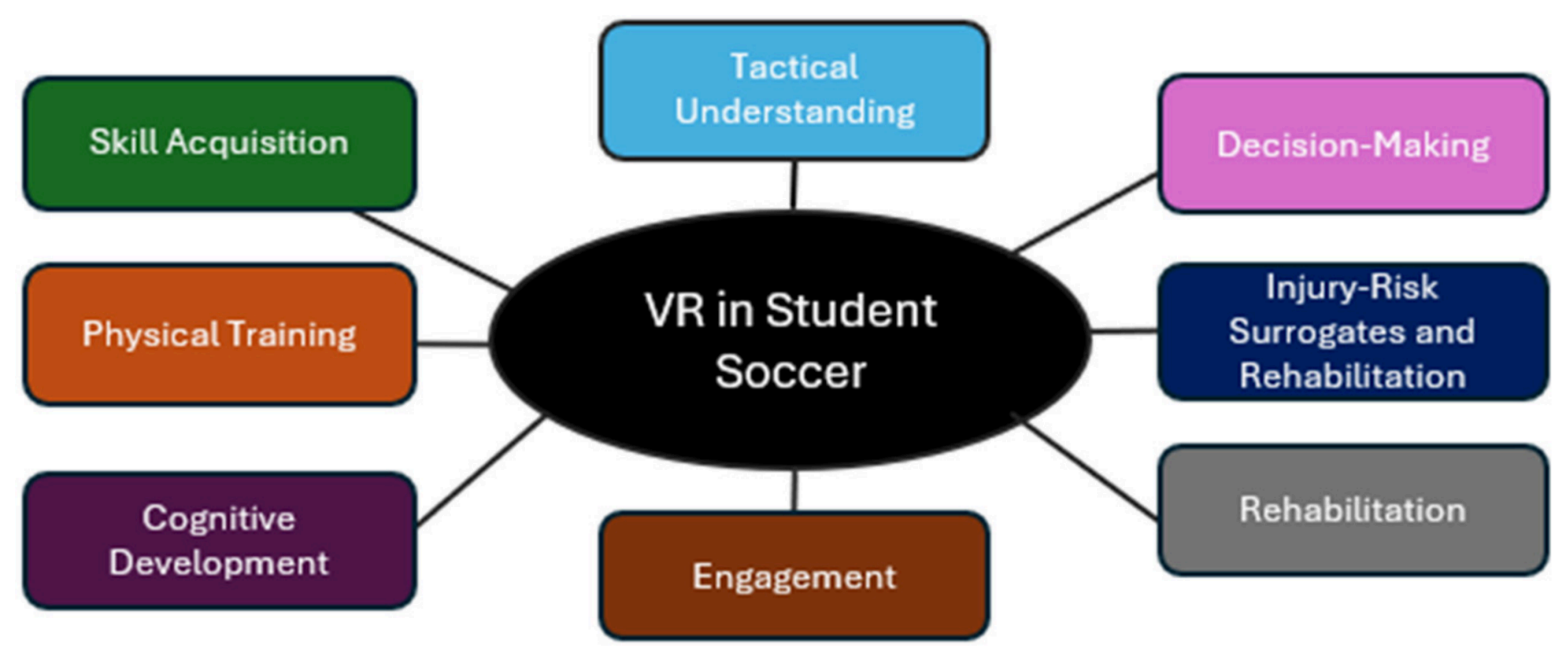
Key outcome domains targeted by VR interventions in student soccer, as identified in this systematic review.

These evidence categories were analyzed separately. Findings from cross-sectional expertise comparisons were interpreted as evidence of discriminative, construct, or predictive validity and were not used to support claims that VR training improves soccer performance. Within each evidence category, the relevant physical, tactical, cognitive, and educational outcomes are summarized are summarized in Figure 3.

Across the included literature, VR was used either as an intervention platform or as an assessment environment. Intervention studies exposed participants to repeated VR training sessions and evaluated changes over time or differences relative to a comparator. In contrast, assessment studies used one-time or short-duration VR tasks to measure expertise-related differences in scanning, attention, gaze behavior, decision-making, or tracking performance. Because these designs address different research questions, their findings are presented separately below. The first stage is task and scenario design. Players are placed in an immersive training environment delivered through an HMD, where they complete controlled drills that reproduce soccer-relevant demands such as scanning for teammates and opponents, selecting passing options under time pressure, orienting the body before receiving the ball, reacting to visual or auditory cues, or executing footwork while maintaining postural control. Depending on the study, these scenarios are tuned towards different outcomes: perceptual-cognitive skills (anticipation, attention, and decision-making) [18,19,20,21,22], neuromuscular control [14,15,16] situational awareness in match-like contexts [4], and psychological engagement and motivation [3,13].

The second stage is training exposure. VR sessions are delivered either as stand-alone sessions (in a lab or dedicated VR room) or as an integrated block within the normal training schedule (for example, appended to weekly academy practice or to a physical education class). Some studies [5,18,22] use short, single-session assessments to quantify perceptual-cognitive skill profiles such as scanning frequency, tracking ability, or inhibitory control. Others use multi-week intervention blocks in which players repeatedly perform VR-based drills designed to improve sensorimotor control, visual exploration, decision-making under pressure, or perception–action coupling in game-like situations [13,19,20,21]. In several instances, the VR content adapts dynamically to the learner. Task difficulty, such as the speed of moving targets, the number of simultaneous stimuli, the number of decision alternatives, and the available response time, increases progressively as the player’s performance improves. This adaptive progression is used to maintain cognitive load, sustain motivation, and approximate the pace and complexity of competitive play [19,20].

The third stage is outcome assessment. Performance is typically captured at two levels. At the in-VR level, the system automatically logs metrics such as response time, decision accuracy, tracking success, scanning rate, pass selection quality, and stability or control during virtual footwork and body positioning tasks [18,20,22]. These metrics allowed frame-by-frame analysis of perceptual, cognitive, and motor behavior in a way that is difficult to obtain during live match play [19,21]. At the off-VR level, studies often collect complementary measures to evaluate transfer and ecological relevance. These include neuromuscular or sensorimotor control tests; balance and coordination assessments; small-sided game statistics such as pass completion rate and tactical execution quality; self-reported motivation and perceived effort ratings; and, in some cases, neurocognitive or physiological indicators such as reaction time and accuracy in a visuomotor Go/No-Go task or preparatory brain activity recorded via electroencephalography (EEG) [1,2]. Taken together, these in-VR and off-VR outcomes serve to evaluate whether VR-based training produces meaningful gains in physical control, tactical awareness, decision-making efficiency, attention regulation, and training motivation across realistic soccer contexts.

Finally, studies evaluated the effectiveness of the intervention within a defined developmental context. Most studies compare VR-based training against a control or alternative approach, such as standard technical–tactical drills, traditional board, video review, or a non-immersive desktop version of the task. Others take a profiling approach, comparing performance across expertise levels (for example, academy versus novice players) to test whether VR can distinguish tactical understanding, perceptual-cognitive efficiency, or neuromuscular control in a way that aligns with on-field status. This comparison step is critical in determining not only whether VR “works,” but also for whom it is most effective (for example, older versus younger players; academy versus recreational players) and in which specific target domain (for example, decision-making versus neuromuscular stability).

### 3.1. Study Characteristics, Evidence Purpose, and VR System Classification

The 20 included studies used heterogeneous VR displays, tracking arrangements, participant populations, research designs, and outcome measures. To support appropriate interpretation, each study was classified according to its primary evidence purpose rather than being treated uniformly as a training intervention.

Training-effectiveness studies used randomized, controlled, quasi-experimental, or repeated-exposure designs to examine whether VR practice produced changes in physical, technical, tactical, perceptual-cognitive, or motivational outcomes. Assessment-validity studies used cross-sectional, expertise-comparison, classification, or prospective profiling designs to determine whether performance within a VR task distinguished player level, reflected established expertise-related differences, or predicted later competitive progression. Rehabilitation and injury-related studies focused on balance, postural control, neuromuscular coordination, heading rehearsal, joint stability, or return-to-play-related mechanisms. Engagement and educational studies assessed motivation, enjoyment, participation, perceived effort, usability, or classroom applicability.

This distinction is important because cross-sectional differences between expert and novice players do not establish that VR exposure caused performance improvement. Accordingly, expertise-comparison studies were interpreted only as evidence that a VR task may possess discriminative, construct, or predictive validity. Training effectiveness was inferred only from studies that evaluated change after repeated VR exposure using a pre–post, controlled, or comparison design.

The included studies also used technically different display and tracking configurations. Head-mounted displays included systems from the HTC Vive, Oculus/Meta, and Valve families; however, these devices were not treated as technically equivalent. Oculus Quest 2 was classified as a camera-based six-degrees-of-freedom tracking system. HTC Vive Pro and Valve Index were described as using SteamVR external base-station tracking only when this configuration was explicitly reported in the source study. When the original article did not provide sufficient technical detail, the system was recorded as “HMD-based positional tracking; exact configuration not reported” rather than assigning a tracking architecture from the commercial device name alone.

Projection-based systems, including Helix-Arena, were classified as 360° immersive projection environments rather than as tracking systems. The display environment and the participant-response or interaction device, such as a handheld HTC Vive controller, were recorded separately. Handheld controllers were therefore not described as independent tracking systems unless the original article explicitly reported their role in positional tracking. Similarly, SMI BeGaze was classified as eye-movement analysis software rather than as a physical eye-tracking sensor [4,5,6,15,21,22]. The actual eye-tracker model, display configuration, sampling rate, and calibration procedure were reported separately when available and recorded as “not reported” when these details were absent. This source-faithful classification was applied consistently throughout the study-level tables and narrative synthesis.

For interpretive clarity, “student and youth soccer” in the following subsections includes school-aged players, academy players, and university or collegiate athletes. A small number of studies involving recreational adults or adults with chronic ankle instability were retained only when the VR task addressed soccer-specific mechanisms relevant to youth safety, rehabilitation, or return-to-play preparation. These adult studies were treated as indirect developmental-transfer or rehabilitation evidence and were not used to draw direct conclusions about training effectiveness in student soccer populations.

The domain-specific findings are presented according to evidence purpose and application type. Training-effectiveness findings are organized into physical and sensorimotor training, tactical and perceptual-cognitive training, and technical skill and imagery training. Assessment studies are presented separately under discriminative, construct, or predictive validity. Rehabilitation and injury-related findings are distinguished from general physical training, while engagement, usability, and educational outcomes are synthesized independently from athletic-performance outcomes.

The extracted evidence is presented in two main tables and one detailed Supplementary Table S3. Here, Table 1 summarizes study and population characteristics, including country, participant classification, sample size, age, soccer context, study design, comparator, and primary evidence purpose. Table 2 presents the principal outcomes and quantitative findings, including measurement instruments, assessment setting, follow-up duration, exact statistical results, effect estimates, confidence intervals, and evidence interpretation. Detailed study-level information regarding VR display environments, positional-tracking configurations, interaction devices, auxiliary sensors, analysis software, and technical parameters is provided in Supplementary Table S3.

VR display, positional-tracking, interaction-device, and auxiliary-sensor classifications were based only on technical information explicitly reported in the original studies. Commercial headset names were not used to infer unreported tracking architectures. SMI BeGaze was classified as eye-movement analysis software rather than as a physical eye-tracking sensor, and Helix-Arena was classified as a 360° immersive projection environment rather than as a tracking system. “Not reported” indicates that the source article did not provide sufficient technical information.

### 3.2. Risk of Bias and Methodological Quality

Study-level methodological assessments are described narratively in this section, with detailed domain-level judgments provided in Supplementary Tables S2.1–S2.4. Nine studies were assessed using RoB 2, six non-randomized intervention studies were assessed using ROBINS-I, and five cross-sectional or profiling studies were assessed using a Downs–Black-based appraisal.

Based on the information available in the published reports, none of the randomized studies could be confidently classified as low risk across all RoB 2 domains; most raised some concerns because allocation concealment, missing-data handling, assessor blinding, or selective-reporting procedures were insufficiently described. The non-randomized intervention evidence generally showed moderate-to-serious concern, primarily because of possible confounding, unclear allocation procedures, and insufficiently described comparators. Cross-sectional and profiling studies were principally limited by non-random group selection, expertise- and age-related confounding, and small samples in several studies. These methodological limitations were considered when interpreting the findings, particularly for claims concerning training effectiveness, real-world transfer, and injury-related benefits.

### 3.3. Training Effectiveness

Training effectiveness was evaluated only in studies that delivered repeated VR exposure and assessed within-participant change, between-group differences, or both. Cross-sectional expertise-comparison studies were excluded from this effectiveness synthesis because they did not manipulate VR training exposure. The intervention evidence included physical and sensorimotor training, tactical and perceptual-cognitive training, technical skill practice, and VR-supported imagery.

#### 3.3.1. Physical and Sensorimotor Training

Five studies examined whether repeated VR exposure improved physical control, sensorimotor performance, or movement-related soccer skills. Ref. [14] evaluated a structured immersive sensorimotor program in collegiate soccer players using a quasi-experimental controlled design. The intervention targeted balance, postural stability, cervical control, coordination, and oculomotor function. Compared with usual strength and conditioning, the VR group showed improvements in neuromuscular control and performance on the trained sensorimotor tasks. However, the study mainly reported surrogate indicators of movement readiness and injury-related control rather than actual injury incidence. Accordingly, its findings support possible short-term improvements in sensorimotor function but do not establish that VR reduces injuries.

Ref. [15] examined short-term home-based VR heading practice in recreational soccer players using a randomized controlled design. Participants assigned to VR heading practice improved heading accuracy, goal-scoring success, and self-reported confidence relative to a no-practice control group. Because the comparator did not receive an active heading intervention, the observed differences cannot be attributed exclusively to the immersive characteristics of VR and may partly reflect general practice effects. The study also involved recreational adults and should therefore be interpreted as indirect evidence for student and youth soccer.

Ref. [25] compared VR-based heading practice with physical ball practice, lightweight-ball practice, combined training conditions, and a control or comparison condition over eight weeks. VR practice improved heading accuracy, indicating that selected timing and directional components of the skill could be rehearsed in an immersive environment. However, physical ball contact remained important for developing heading strength and ball-flight distance. These findings suggest that VR may function as a complementary method for technical rehearsal rather than a complete replacement for physical soccer practice. Outside the VR literature, IMU-based motion analysis has independently validated kicking kinematics in football, reinforcing the biomechanical relevance of the outcomes VR heading and kicking studies aim to approximate [34].

Ref. [28] evaluated an eight-week VR-based multimodal training program targeting balance and agility in young male soccer players (11–13 years) using a randomized controlled design against usual soccer training. The VR group showed improvement on the trained balance and agility measures, consistent with the pattern of short-term sensorimotor gains observed in [14]. Ref. [30] examined VR-assisted coaching against blended, traditional, and self-paced online conditions in adolescent female footballers (14–18 years) at a football training academy, reporting improvements in physical outcomes for the VR-assisted condition relative to the comparison arms; corresponding psychological and motivational findings from this study are discussed in Section 3.6.

Taken together, the physical and sensorimotor intervention studies provide preliminary evidence that repeated VR practice may improve selected aspects of balance, neuromuscular control, coordination, and heading accuracy. Nevertheless, the evidence remains limited by heterogeneous populations, small samples in some studies, short intervention periods, incomplete reporting of effect sizes, and reliance on surrogate outcomes rather than injury incidence or full-match performance. Ref. [16] was not included in this subsection because it involved participants with chronic ankle instability and is therefore discussed primarily under rehabilitation.

#### 3.3.2. Tactical and Perceptual-Cognitive Training

Five intervention studies examined whether repeated VR training improved scanning, attention, tactical decision-making, anticipation, passing behavior, or perceptual-cognitive processing. These studies provide intervention evidence because participants completed repeated VR sessions and were assessed longitudinally or compared with control or alternative-training conditions.

Ref. [21] examined systematic perceptual-cognitive training using 360° VR. The study reported improvement in the trained perceptual-cognitive task; however, evidence of transfer to broader soccer-specific performance measures was limited. Accordingly, the findings are interpreted as primarily task-specific rather than as confirmed improvement in real-world tactical performance. Because this study used repeated training and control conditions, it contributes to the evidence for tactical and perceptual-cognitive training effectiveness rather than assessment validity. However, the manuscript states that randomization was not specified, which limits confidence in causal attribution.

Ref. [20] used a randomized parallel-group design to compare immersive VR tactical training with video-based instruction in national-level youth players. The VR group showed larger improvements in small-sided-game decision accuracy and visual-search efficiency, including fixation-related measures, than the video group. These findings provide controlled evidence that immersive first-person training may improve selected tactical and perceptual-cognitive outcomes beyond conventional video instruction. Nevertheless, the small sample and limited follow-up restrict conclusions regarding durability and transfer to full-match performance.

Similarly, improved performance on an in-VR tactical task should not be assumed to represent improved competitive-match performance. Coaches should distinguish between task-specific learning within VR, transfer to a controlled field assessment, transfer to small-sided play, and transfer to full matches. At present, the evidence most strongly supports VR as a supplementary environment for repeated perceptual-cognitive practice and immediate feedback; evidence supporting durable full-match performance improvement remains insufficient.

Ref. [5] evaluated an eight-week randomized cognitive–motor VR intervention in semi-elite youth academy players. The training included repeated Rezzil drills and incorporated visuomotor and electroencephalographic outcomes. Compared with standard training, the VR group showed improvements in response accuracy, reaction-related performance, motor preparation, and anticipatory neural activity. These results suggest that repeated VR exposure may influence both behavioral performance and neurophysiological preparation. However, the reported EEG changes were not accompanied by a corresponding full-match transfer measure, and therefore their practical significance remains uncertain.

Ref. [23] was a pilot intervention involving elite youth academy players. The VR group showed a descriptive increase in pre-reception scanning (+49.3%) compared with a small decrease in controls; however, the between-group effect was not statistically significant (F=2.56, p=0.132, $\eta^{2}=0.155$). Passing success also showed no significant between-group change (F=0.023, p=0.882, $\eta^{2}=0.002$). Therefore, although the scanning effect estimate was relatively large, evidence of transfer to small-sided-game performance remains preliminary. This study is particularly relevant because it assessed behavior outside the trained headset task. However, the small sample, insufficiently described comparator, and incomplete reporting of the intervention platform reduce the certainty of the findings.

Ref. [24] used a seven-week randomized controlled design to compare SensiballVR-based cognitive and technical drills with traditional football training. The VR group showed meaningful improvement in scanning behavior, while passing outcomes showed more exploratory or less conclusive improvement. The study therefore supports a possible effect of VR on visual exploration and perceptual-cognitive behavior, but the evidence for broader technical or match-performance improvement remains limited.

Overall, the tactical and perceptual-cognitive intervention studies suggest that repeated VR training may improve scanning, attention, decision accuracy, anticipation, visual-search behavior, and selected passing-related outcomes. The strongest evidence comes from controlled studies comparing VR with video instruction, routine practice, or another active condition. However, most outcomes were measured immediately after training, within VR, in laboratory tasks, or during small-sided games. Long-term retention and transfer to full competitive match performance remain insufficiently established.

#### 3.3.3. Technical Skill and Imagery Training

Four studies examined VR as a method for developing technical soccer skills or supporting skill acquisition through imagery-based practice.

Ref. [15] evaluated home-based VR heading practice and reported improvements in heading accuracy, goal-scoring success, and confidence compared with a no-practice control. These findings indicate that repeated immersive rehearsal may improve selected components of heading performance. However, because the control group did not complete an active alternative practice condition, it remains unclear whether the benefits were specific to VR or reflected the general effect of additional practice. The adult recreational sample also limits direct generalization to student and youth populations.

Ref. [25] compared several heading-training approaches, including VR-based practice, conventional soccer-ball practice, lightweight-ball practice, combined conditions, and a control or comparison condition. VR training improved heading accuracy, whereas ball-contact training remained important for increasing heading strength and ball-flight distance. The findings indicate that VR may be useful for practicing timing, orientation, and directional accuracy while reducing repeated physical head impacts, but it should be considered an adjunct to rather than a replacement for physical technical training.

Ref. [27] investigated a 12-week smartphone-based VR imagery intervention in youth football players. Participants were allocated to VR imagery, conventional imagery-script, or control conditions. The VR imagery group showed greater improvement in football-kicking performance than the comparison groups and also demonstrated favorable motivation-related outcomes. These findings suggest that immersive imagery may support both technical skill acquisition and psychological engagement. However, the smartphone-based system provided a lower level of interaction and motion tracking than fully interactive HMD systems, and the study did not establish whether the gains transferred to competitive match performance.

Ref. [29] examined the integration of 3-D VR immediately prior to small-sided games in young soccer athletes, reporting improvements in technical performance relative to a small-sided-games-only comparator. Because the reported population was described as “young soccer athletes” without explicit confirmation of enrollment in a school, academy, or university pathway, this study’s structural eligibility should be verified against full-text recruitment details rather than assumed from the abstract alone.

Collectively, the technical skill and imagery studies indicate that VR may support the rehearsal of heading accuracy, kicking performance, timing, and motor imagery. The findings are most appropriately interpreted as evidence for complementary practice. Physical interaction with the ball, active comparison conditions, and ecologically valid transfer assessments remain necessary to determine whether VR-based technical gains persist and generalize to real soccer performance.

### 3.4. Assessment and Discriminative Validity

Five studies primarily evaluated VR as an assessment or profiling environment rather than as a training intervention. Refs. [6,18,19,26] compared performance across expertise, age, or skill groups, while [22] examined whether a VR decision-making test differentiated competitive levels and predicted later progression. Collectively, these studies indicate that selected VR tasks may be sensitive to expertise-related differences in visual search, multiple-object tracking, tactical decision-making, gaze efficiency, and attention allocation.

**Figure 4:**
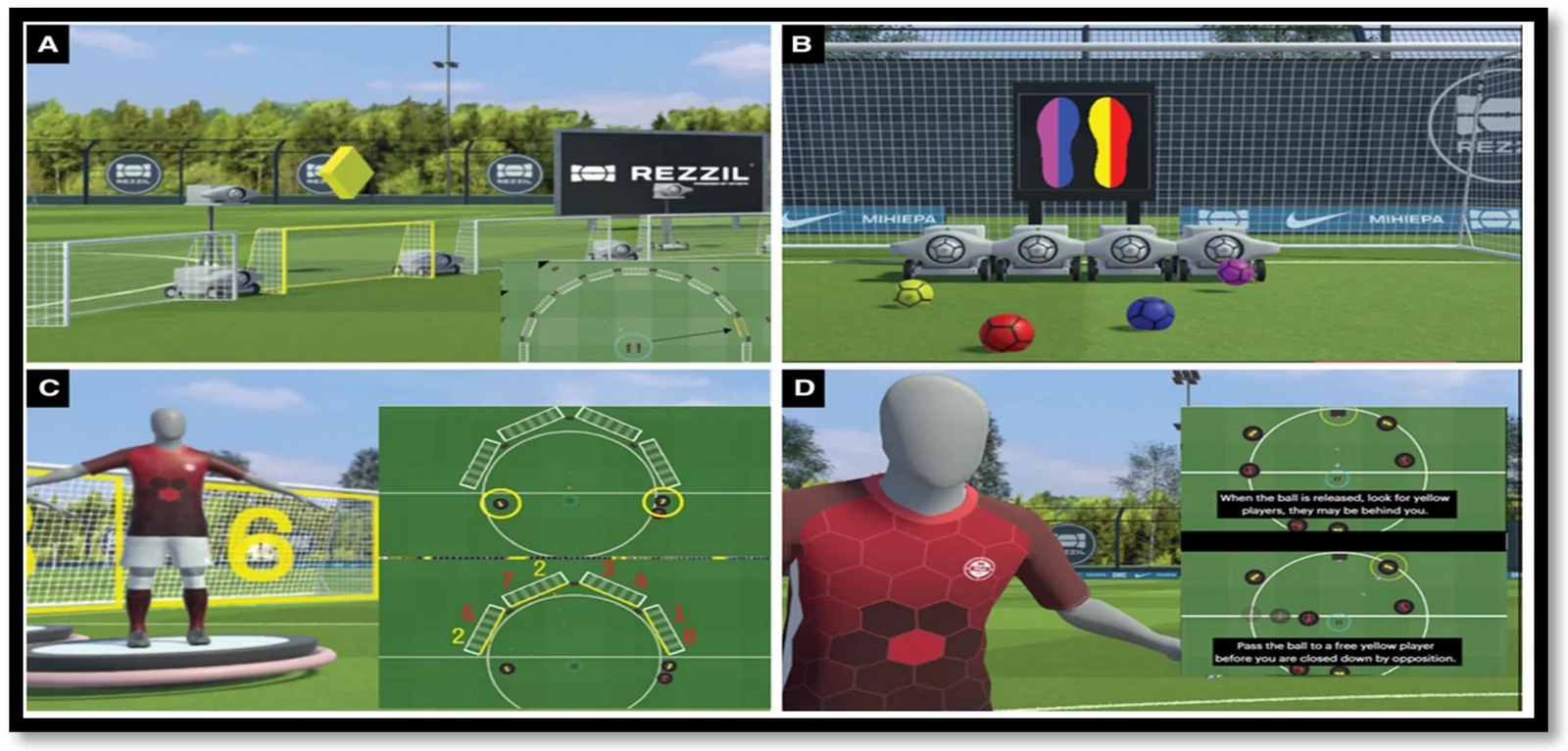
Representative soccer-specific virtual-reality assessment tasks implemented in the Rezzil/MiHiepa environment: (**A**) Rondo Scan; (**B**) Color Combo; (**C**) Shoulder Sums; and (**D**) Pressure Pass. The panels illustrate task scenarios involving visual scanning, target recognition, spatial awareness, and decision-making. Adapted/reproduced from Ref. [21], subject to the original publisher’s permission requirements.

These results should not be interpreted as evidence that VR training improves performance. Because participants were not assigned to repeated VR training and assessed for intervention-related change, the observed differences may reflect prior playing experience, age, maturation, competitive level, or other pre-existing characteristics. The findings therefore support discriminative, construct, or predictive validity of the VR assessment tools, subject to the methodological limitations of the individual studies. Representative examples of the soccer-specific VR assessment tasks used in the Rezzil/MiHiepa environment are shown in Figure 4.

### 3.5. Rehabilitation and Injury-Related Applications

Rehabilitation evidence was distinct from general training-effectiveness evidence. Ref. [16] evaluated VR-based balance training in adults with chronic ankle instability and reported improvements in postural control, dynamic balance, muscle activation, and perceived ankle stability relative to traditional balance exercises. Because the participants were adults with a clinical condition, this study was treated as indirect rehabilitation evidence rather than direct evidence from student or youth soccer.

Refs. [14,15,25] examined neuromuscular control or safer rehearsal of heading-related skills. These studies measured performance or surrogate indicators related to injury risk but did not evaluate injury incidence, reinjury, or formal return-to-play outcomes. They therefore support possible injury-related applications rather than confirmed injury-prevention effectiveness.

### 3.6. Engagement, Usability, and Educational Outcomes

Engagement, usability, and educational outcomes were evaluated separately from athletic-performance outcomes. Ref. [13] examined VR-supported soccer activities in elementary-school physical education and reported motivation, attention, enjoyment, participation, and system-mediated task feedback. Ref. [4] directly compared VR and non-VR task modes in an adult sample and assessed hedonic motivation and perceived effort using five-point rating scales. Ref. [22] reported immersion ratings and qualitative interview responses in a VR decision-assessment context, while [27] included motivation-related outcomes alongside football-kicking performance. Ref. [29] also reported engagement-related outcomes, including intention to continue exercising and teamwork, alongside its technical-performance findings. Ref. [30] reported favorable psychological and motivational outcomes for the VR-assisted coaching condition in adolescent female footballers, complementing the physical findings summarized in Section 3.3.1.

The available evidence therefore addressed only a limited subset of HCI-related constructs. Reported measures primarily concerned motivation, enjoyment, perceived effort, immersion, participation, and basic feasibility. The included studies did not systematically evaluate presence, embodiment, cognitive workload using validated multidimensional instruments, controller learnability, interaction errors, accessibility, cybersickness, physical comfort, system usability using standardized scales, or sustained user experience over repeated sessions.

Accordingly, these findings support only preliminary conclusions regarding engagement and selected aspects of usability. They do not constitute a comprehensive evaluation of the interaction design or user experience of VR systems in student soccer. Ref. [4] also involved general adults and was therefore treated as indirect usability and feasibility evidence rather than direct evidence from student or youth soccer.

### 3.7. Transfer, Retention, and Real-World Performance

Most intervention studies evaluated immediate post-intervention or in-VR outcomes. Evidence of transfer beyond the trained environment was limited. Ref. [23] reported changes in scanning and pass selection during small-sided play, while some other studies included laboratory or controlled field measures. However, few studies assessed full-match performance, and long-term retention was rarely examined. Accordingly, improvements in VR task scores should not automatically be interpreted as durable improvements in real-world soccer performance.

## 4. Discussion

### 4.1. Principal Findings

The present review extends a growing body of domain-specific VR systematic reviews in sports science. A recent review by Ghazanfar et al. [10] examined VR and exergaming in tennis, reporting positive effects on human performance, engagement, and skill transfer in student and youth player populations. The findings of the present soccer review align with those tennis-domain conclusions in two key respects: VR consistently supports engagement and perceptual-cognitive outcomes across both sports, and transfer to live performance remains the central open question in both domains. The soccer context, however, introduces distinct demands not present in tennis VR research—specifically, the team-tactical dimensions of scanning, spatial awareness, and multi-player decision-making—as well as a broader population range spanning school physical education, youth academies, and university programs. The present review is, to our knowledge, the first to characterize VR sensing hardware configurations alongside developmental outcomes specifically within student and youth soccer, thereby extending prior domain-specific VR reviews into a new sport and population context.

This systematic review brings together research evidence from 20 included studies on the use of VR in student and youth soccer across the five developmental domains introduced in Section 3.1. First, intervention studies examined whether repeated VR training improved physical, technical, tactical, perceptual-cognitive, or motivational outcomes. These studies provide preliminary evidence of short-term training effects, although the findings are constrained by small samples, heterogeneous comparators, and limited follow-up. Second, assessment and profiling studies examined whether VR task performance differed across expertise, age, or competitive level. These studies indicate that certain VR tasks may possess discrimination, construct, or predictive validity, but they do not establish that VR training causes performance improvement.

Rehabilitation and injury-related evidence was more limited and included indirect evidence from adult or clinical populations. From an interaction perspective, the evidence was considerably narrower than the performance and perceptual-cognitive evidence. Only a small subset of studies reported usability-related outcomes, primarily hedonic motivation, perceived effort, immersion ratings, participation, or qualitative experience. Core HCI constructs—including presence, embodiment, interaction efficiency, error recovery, cognitive workload, accessibility, learnability, comfort, cybersickness, and sustained user experience—were not assessed consistently. Therefore, the review does not support broad conclusions about the overall HCI quality of the included systems.

A critical distinction is required between improvement within the trained virtual environment and transfer to real soccer performance. Most intervention studies reported immediate post-training changes in VR task scores, laboratory measures, controlled technical tests, or small-sided-game behaviors. Such findings indicate near transfer to similar tasks or settings but do not necessarily demonstrate far transfer to competitive match performance. Among the studies included, evidence extending beyond the trained VR task was limited. Ref. [23] assessed scanning and pass-selection behavior during small-sided play, and selected studies used laboratory or controlled-field outcomes; however, these settings remain less complex than full competitive matches. No consistent evidence established sustained improvement in match-level tactical behavior, technical execution, or season-long performance. Long-term retention was also rarely assessed, as most studies used immediate post-intervention testing without delayed follow-up.

Rehabilitation and injury-related evidence was limited and should be interpreted cautiously. The included studies reported improvements in outcomes such as balance, postural control, neuromuscular activation, heading accuracy, and confidence. These are performance, rehabilitation, or injury-risk surrogate measures rather than direct injury outcomes. None of the included studies demonstrated that VR reduced injury incidence, prevented reinjury, shortened return-to-play time, or improved formal medical-clearance outcomes. Therefore, the evidence supports the potential use of VR for controlled rehearsal and rehabilitation-related training but not confirmed injury-prevention or return-to-play effectiveness.

Fewer studies directly examine student-centered engagement outcomes. School-based physical education studies report increased attention, motivation, and willingness to participate when VR is integrated into soccer instruction, indicating that VR has pedagogical value beyond pure performance metrics [13]. Finally, it is important to note that most reported gains are short-term (immediate post-test or brief retention) and are derived from controlled VR environments or small-sided games, rather than full match play. Long-term skill retention and real-world transfer to full-match performance remain largely untested, primarily due to the lack of longitudinal follow-up, inconsistent assessment protocols, small sample sizes, and the limited use of ecological outcome metrics in existing studies.

### 4.2. Comparison with Prior Reviews

Beyond restricting scope to student and youth populations, this review contributes a methodological framework not present in prior sport-VR syntheses: a structured classification of sensing hardware (display type, motion-tracking modality, auxiliary peripherals, and reported technical parameters; Section 3.1 and Supplementary Table S3) that is cross-referenced against outcome domain and evidence quality. Prior reviews [10,12,35] report intervention outcomes without systematically characterizing the sensing configurations that produced them. By coding reported sensing configurations alongside outcomes, this review identifies a hardware–outcome relationship that was not made explicit in prior sport-VR reviews [8,11,13].

In contrast, the present review restricts its scope to studies published between January 2020 and August 2026, a period during which head-mounted VR systems became more affordable, tracking accuracy and responsiveness improved, and schools and academies began using VR as part of structured training rather than as a novelty. This review specifically examines student and youth soccer within schools, academies, and university settings, synthesizing findings across five developmentally relevant domains. By organizing the evidence along these domains, this review extends the prior literature by showing that VR in student soccer is being investigated not only for performance-related applications but also as a potential educational medium. VR is being used to support decision-making under pressure, attention control, controlled neuromuscular rehearsal, motivation to participate in practice, and rehabilitation-related readiness. Positioning VR as both a training technology and a learning environment addresses a gap left by earlier sport-VR syntheses while also highlighting its direct relevance to curriculum design and player development in youth soccer.

### 4.3. Strengths and Limitations

A major strength of this review is its focused scope; to our knowledge, this review is the first to integrate a structured classification of VR sensing and tracking configurations with evidence purpose and developmental outcomes specifically in student and youth soccer. By integrating evidence across five domains (physical training, tactical understanding, injury-related and rehabilitation outcomes, engagement and motivation, and perceptual-cognitive development), the review provides a structured view of how VR is being used not just for performance gains but for education, readiness, and return-to-play support.

A major limitation concerns the ecological level and timing of outcome assessment. Most reported improvements were measured immediately after the intervention and within the trained VR environment, in laboratory tasks, or in controlled technical assessments [13,14,15,16,20,21,23,24,25]. A smaller number of studies examined behavior during small-sided games, which provides preliminary evidence of transfer beyond the headset but does not reproduce the tactical complexity, fatigue, opponent interaction, and temporal demands of full competitive matches [23,24]. Consequently, in-VR improvement, laboratory transfer, controlled-field transfer, small-sided-game transfer, and full-match transfer should not be treated as equivalent levels of evidence. Delayed retention testing was uncommon, and the available literature does not establish whether reported gains persisted for weeks or months after training ended. The review therefore cannot determine whether VR produces durable changes in competitive soccer performance.

The injury-related evidence has similar limitations. Included studies assessed balance, postural control, muscle activation, heading performance, confidence, and other surrogate measures associated with movement readiness or injury risk [14,15,16,25]. They did not directly measure injury incidence, reinjury frequency, days lost from training or competition, time to return to play, or successful medical clearance. Accordingly, improvements in these surrogate measures should not be interpreted as evidence that VR prevents injury or accelerates return to sport.

Several limitations should be considered when interpreting this review. First, the evidence base was small, comprising 20 included studies, and the participant populations were heterogeneous. Although the primary scope concerned school-aged, academy, university, and collegiate soccer participants, a small number of studies involved recreational adults or adults with chronic ankle instability [15,16]. These studies were retained only as indirect mechanism-level evidence for rehabilitation, neuromuscular control, heading rehearsal, or return-to-play-related applications and were not used to draw direct conclusions about training effectiveness in student or youth soccer. Nevertheless, combining evidence across school, academy, collegiate, recreational-adult, and clinical populations limits population-level comparability and reduces the generalizability of the synthesis.

Second, the included studies were methodologically heterogeneous. Designs included randomized controlled trials, quasi-experimental studies, short-term pilot interventions, cross-sectional expertise comparisons, and prospective profiling studies [6,13,14,15,16,18,20,21,22,23,24,25,26]. Considerable variation was also present in intervention duration, session dosage, control conditions, immersion level, outcome instruments, and evidence purpose. These differences prevented meaningful statistical pooling and limited direct comparison across studies.

Third, most intervention studies used immediate post-intervention or single-session assessments, and delayed follow-up was uncommon [13,14,15,16,20,21,23,24,25]. Consequently, the review cannot determine whether reported improvements persisted for weeks or months after VR exposure ended. Most outcomes were assessed within VR, in laboratory tasks, in controlled technical tests, or during small-sided games. Evidence of durable transfer to full competitive-match performance, injury reduction, reinjury prevention, or return-to-play outcomes remains limited.

Fourth, publication bias may have influenced the evidence base because most included studies reported positive or favorable findings, whereas relatively few reported null, negative, or adverse outcomes. Because the small number and methodological diversity of the included studies prevented meaningful formal assessment using funnel plots or related statistical methods, publication bias could not be quantified. Language bias is also possible because eligibility was restricted to English-language publications. Therefore, relevant studies published in other languages may have been missed.

Fifth, the review protocol was registered retrospectively on the Open Science Framework rather than prospectively before study identification, screening, and data extraction. Although retrospective registration improved transparency by making the review methods publicly available, it could not eliminate the possibility that eligibility, outcome-classification, or synthesis decisions were influenced by knowledge of the identified literature. The retrospective registration should therefore not be interpreted as equivalent to prospective protocol registration.

Sixth, technical reporting was incomplete across much of the included literature. Important specifications—including display resolution, motion-to-photon latency, frame rate, field of view, tracking degrees of freedom, sensor sampling rate, calibration procedures, tracking accuracy, and software versions—were frequently absent. These omissions limited reproducibility and prevented evaluation of whether tracking fidelity, display quality, or sensor configuration moderated intervention outcomes. Missing technical information was recorded as “not reported” rather than inferred from commercial device names, and the available study-level specifications are summarized in Supplementary Table S3.

A further limitation concerns the analysis of human–system interaction. Although some included studies reported motivation, perceived effort, immersion, participation, or user-experience outcomes [3,4,13,22,27], these measures were heterogeneous and were not based on a consistent HCI evaluation framework. Presence, embodiment, learnability, interaction efficiency, cognitive workload, accessibility, cybersickness, physical comfort, and standardized usability were rarely assessed. Consequently, the review could not compare the HCI quality of different VR platforms or determine how interaction design influenced training adherence, learning, or performance outcomes.

Additional review-level limitations should also be acknowledged. The original database-search execution dates and database-specific retrieval counts were not retained, limiting full retrospective reproducibility of the identification stage despite the reconstructed strategies provided in Supplementary Table S1. Inter-rater agreement for screening and extraction was not quantified using Cohen’s κ or a comparable statistic. In addition, most samples were predominantly or exclusively male; the search update added one all-female adolescent cohort [30], but generalizability to female players, mixed-gender educational settings, goalkeepers, referees, and other soccer roles remains limited.

### 4.4. Implications for Practice

The findings suggest several possible applications for teachers, coaches, clinicians, and curriculum designers who are considering VR in student soccer. However, these applications should be interpreted cautiously because the available evidence is based on a small and heterogeneous group of studies, and most practical outcomes were assessed only over short periods.

In school-based physical education, one study reported greater motivation, attention, enjoyment, and participation during VR-supported soccer activities than during conventional physical education [13]. System selection should therefore not be based solely on performance or sensing capability; schools and academies should also consider usability, physical comfort, accessibility, cybersickness, setup complexity, interaction learnability, and supervision requirements, although comparative evidence for these factors remains insufficient. These findings suggest that VR may be used as a supplementary instructional medium to support engagement under selected classroom conditions. VR platforms may also provide immediate task feedback when such functionality is incorporated into their design. Nevertheless, the included studies did not establish broad educational effectiveness, long-term learning gains, improved curriculum outcomes, or successful large-scale implementation.

VR may also offer opportunities for more individualized or inclusive task delivery by allowing task difficulty, physical demand, and feedback to be adjusted. However, inclusive participation, accessibility for students with disabilities, mixed-ability learning, and educational equity were not directly assessed. These should therefore be regarded as plausible applications requiring dedicated evaluation rather than as confirmed benefits.

At the academy and university levels, VR platforms have been employed to recreate match-pressure tactical scenarios and to assess or train perceptual-cognitive skills, including scanning, anticipation, and passing decisions under time constraints [18,22,23]. These applications may support performance coaching in two ways: (i) by allowing staff to personalize feedback using objective and repeatable diagnostic tasks, and (ii) by providing athletes with high-repetition tactical exposure without the physical cost of full-intensity match play.

However, improved performance within an in-VR tactical task should not be assumed to represent improved competitive-match performance. Coaches should distinguish between task-specific learning inside VR, transfer to a laboratory or controlled-field assessment, transfer to small-sided play, and transfer to full competitive matches. At present, the evidence most strongly supports VR as a supplementary environment for repeated perceptual-cognitive practice, immediate feedback, and controlled rehearsal. Evidence of durable improvement in full-match tactical or technical performance remains insufficient.

Rehabilitation-related studies suggest that VR may provide a controlled environment for practicing balance, postural control, neuromuscular coordination, and selected heading-related skills. These applications may be useful as complementary training or rehabilitation activities because task difficulty can be progressively adjusted and repeated under supervised conditions. However, the reviewed evidence does not demonstrate reduced injury incidence, lower reinjury risk, faster return to play, or improved medical-clearance outcomes. Coaches and clinicians should therefore interpret VR as a possible adjunct to conventional rehabilitation and injury-risk management rather than as a validated injury-prevention or return-to-play intervention.

Because VR sessions can be structured, supervised, and progressively adapted in a controlled environment, they are particularly appealing for athletic training rooms and recovery clinics working with student-athletes. Looking forward, as VR hardware becomes more portable and adaptive training systems become more common, these platforms could be woven into regular teaching and training rather than treated as a novelty. In that model, VR is not just a “fun drill” but an instructional space where athletes rehearse decision-making, receive immediate feedback, and link cognitive understanding to physical execution. This aligns VR with core pedagogical goals in physical education and academy development: guided discovery, scenario-based reasoning, and progressive autonomy in tactical decision-making.

Large-scale educational use cannot currently be recommended based on the included evidence. Most studies were conducted in controlled, small-sample, or short-term settings and did not evaluate institutional cost, equipment availability, classroom space, teacher training, technical support, cybersickness, maintenance burden, data governance, or sustained student use. Accordingly, school- or academy-wide implementation should be considered a future possibility rather than an evidence-supported current benefit.

### 4.5. Future Directions

#### 4.5.1. Standardization of VR Training Protocols in Student Soccer

Future research in VR for student and youth soccer should first establish standardized, reproducible intervention protocols. Currently, studies vary significantly in session duration, immersion level, target focus, and the type and quality of comparison groups. These differences include fully immersive HMDs versus panoramic 360° projection systems, perceptual-cognitive versus neuromuscular or motivational outcomes, and active versus passive control conditions. Some studies deliver multi-week VR exposure integrated into physical education or academy training to target perceptual-cognitive, sensorimotor, or decision-making outcomes [5,14,20,21,24], whereas others use single-session perceptual-cognitive profiling in immersive environments [4,18,22]. Reviews of sports-related VR highlight that the heterogeneity in hardware, task design, and reporting styles hampers comparability across studies, limits the accumulation of evidence, and slows adoption in applied settings such as schools and academies [7,35].

To move toward comparable evidence, future VR studies in student soccer should, at minimum, report: (i) training frequency and duration; (ii) the primary intended domain (tactical decision-making, perceptual-cognitive control, neuromuscular stability, injury preparedness, engagement/motivation); (iii) immersion level, latency, and tracking fidelity; (iv) the presence and nature of a control or comparison group (for example, traditional video review, standard on-field practice, or no additional training); and (v) ecological fidelity of the VR scenario such as first-person match context versus abstract cognitive task. Consistent reporting across these dimensions will facilitate cross-study synthesis and enable coaches, physical education teachers, and clinicians to determine whether a given VR protocol functions as decision-making training, stability or coordination training, rehabilitation support, or classroom-based tactical instruction.

#### 4.5.2. Longitudinal Retention, Field Transfer, and Injury Relevance

Future studies should distinguish clearly between task learning inside VR and transfer to authentic soccer performance. At minimum, outcomes should be categorized as: (i) in-VR performance; (ii) laboratory or cognitive-task transfer; (iii) controlled technical or field-test transfer; (iv) small-sided-game transfer; and (v) full competitive-match transfer. Improvements at one level should not be assumed to generalize automatically to the next.

Future controlled trials should incorporate delayed follow-up assessments and ecologically valid soccer outcomes. These should include standardized analysis of small-sided and competitive-match behavior, coded match footage, wearable or positional-tracking data, and exposure-adjusted injury surveillance. Without evidence of retention, full-match transfer, injury incidence, and return-to-play outcomes, VR should remain a complementary training and rehabilitation tool rather than a validated substitute for conventional practice or clinical management.

A second priority is to generate longitudinal evidence on retention and on-field transfer. Most current studies with students and academy players report immediate or short-term improvements directly after VR intervention, including faster option selection, better scanning and anticipation under pressure, improved balance and postural control, and higher-quality passing decisions in simulated match scenarios [15,18,19,20]. Some work suggests that immersive VR may outperform conventional 2D video-based instruction for selected perceptual-cognitive outcomes under time constraints [23]. Selected studies have also used VR for controlled rehearsal of balance, sensorimotor control, and heading-related skills [15,16,17]. However, these studies did not demonstrate reduced injury incidence, lower reinjury risk, or safer return to play; these potential applications therefore require prospective evaluation.

However, two major gaps remain. First, very few studies examine whether these gains are sustained over weeks or months after VR exposure ends. Second, almost none explicitly link VR exposure to (i) behavior in real match play (for example, decision-making success in live small-sided games or competitive fixtures) or (ii) injury incidence, reinjury avoidance, or time-to-clearance in return-to-play pathways for youth athletes. Given the substantial lower-extremity injury burden reported in youth soccer [8], future studies should determine whether controlled VR rehearsal can contribute meaningfully to injury-risk management.

Future trials should therefore incorporate delayed follow-up testing, observational analysis of real competitive contexts (coded match footage or GPS tracking), and basic injury or recovery tracking. Without evidence of retention, transfer, and injury relevance in authentic settings, such as physical education in schools or academy-level competitions, stakeholders are likely to remain cautious about integrating VR into standard development pathways [7,35,36].

#### 4.5.3. Adaptive and Individualized VR Training

A third direction is to evaluate adaptive VR systems that personalize difficulty and cognitive load. Recent soccer-oriented platforms such as CortexVR present first-person, game-like decision tasks—for example, tracking movement of marked players, locating viable passing options, or identifying the ball’s position under pressure. These platforms also dynamically scale visual complexity, speed, and number of simultaneous stimuli to match the athlete’s performance in real time [4]. Parallel work in VR cognitive training, esports, and neurotechnology shows that physiological and neurocognitive signals, including gaze allocation patterns, reaction time in inhibition tasks, heart-rate indicators of arousal, and EEG markers of preparatory attention, can be used to infer cognitive workload. This estimate can then be used to automatically adjust task difficulty so that the learner remains in an optimal challenge zone instead of being underloaded or overwhelmed [36,37,38].

Outside sport-specific contexts, recent work combining EEG sensing with VR-based learning environments in student populations has demonstrated the feasibility of multimodal sensor pipelines for real-time attention and engagement monitoring [39], supporting the extension of similar EEG–VR sensing architectures to adaptive student-soccer training systems.

This approach is particularly relevant in school and academy settings, where athletes of the same chronological age often exhibit significant differences in perceptual maturity, attentional control, decision-making speed, and self-regulation under pressure. Future studies in student soccer should test whether adaptive, biofeedback-informed VR accelerates tactical learning and cognitive control more effectively than fixed-difficulty VR; whether it helps less experienced or slower-developing players “catch up” without overloading them; and whether it sustains engagement and motivation across repeated sessions [4,36,37,38]. Demonstrating scalable individualization will be essential for realistic classroom or academy rollout, where mixed-ability groups are the norm.

#### 4.5.4. Hardware Configuration, Technical Reporting, and Comparative Evaluation

The included studies used heterogeneous display, positional-tracking, interaction, and auxiliary-sensing architecture. HMD-based systems included HTC Vive Pro, Oculus Quest 2, Valve Index, and Oculus Rift devices; however, these systems were not treated as technically equivalent because their tracking arrangements differed. Oculus Quest 2 uses camera-based inside-out six-degrees-of-freedom tracking, whereas HTC Vive Pro and Valve Index generally rely on SteamVR external base-station tracking when this configuration is explicitly reported. The precise tracking arrangement used with Oculus Rift depends on the model and study-specific setup. Because several source articles did not provide sufficient technical detail, tracking architectures were recorded as “not reported” rather than inferred solely from commercial headset names.

Projection-based systems such as Helix-Arena were classified separately as 360° immersive display environments rather than as tracking systems. Handheld controllers or other response devices were documented independently from the display environment. Similarly, motion controllers were not treated as standalone sensing systems unless the source study explicitly described their role in positional or movement tracking. Eye-movement analysis software, such as SMI BeGaze, was also distinguished from the physical eye-tracking hardware used to collect gaze data.

This technical heterogeneity limits comparisons of tracking fidelity, system latency, ecological validity, interaction quality, and outcome performance across studies. Moreover, reporting of frame rate, motion-to-photon latency, field of view, calibration procedures, sensor sampling rate, and tracking accuracy was incomplete in much of the literature. Consequently, the present evidence cannot determine whether one display or tracking architecture produces superior training, assessment, rehabilitation, or engagement outcomes.

Future studies should report display hardware, positional-tracking architecture, interaction devices, auxiliary sensors, calibration procedures, sampling rates, latency, frame rate, and field of view using standardized terminology. Direct within-sample comparisons of HMD-based, projection-based, and desktop systems using equivalent tasks and outcome measures are also needed. Such studies would provide evidence-based guidance for schools and academies selecting VR systems according to immersion, cost, portability, setup burden, cybersickness risk, and intended educational or training purpose.

#### 4.5.5. Usability, Interaction Design, and Accessibility Evaluation

Future research should apply standardized HCI methods to evaluate how athletes, students, teachers, coaches, and clinicians interact with soccer-specific VR systems. At minimum, studies should assess usability, task learnability, interaction errors, cognitive workload, presence, embodiment, comfort, cybersickness, accessibility, satisfaction, and intention to continue use. Validated instruments should be used where appropriate, together with objective interaction measures such as task-completion time, response errors, controller failures, calibration time, dropout, and assistance requirements [40,41].

Interaction design should also be evaluated across age, experience, physical ability, and educational context. Systems designed for children or mixed-ability classes may require simplified controls, adjustable task speed, accessible feedback modalities, seated or low-mobility options, and clear supervision procedures. Comparative studies should determine whether controller-based, body-tracked, gaze-based, projection-based, and HMD-based interfaces differ in usability, cognitive workload, comfort, accessibility, and learning outcomes.

These data are necessary before claims can be made regarding the suitability of VR for routine classroom, academy, or rehabilitation use. Without systematic HCI evaluation, technically sophisticated systems may still be difficult, uncomfortable, inaccessible, or impractical for intended users.

#### 4.5.6. Broadening the Learner and Addressing Feasibility and Ethics

Future research should broaden the conceptualization of the learner and address feasibility, implementation, and ethical governance. Beyond outfield players, immersive VR and 360° video are already being explored for referees and coaches. These applications allow referees to rehearse foul recognition, offside judgment, and positioning at match speed, and allow coaches to guide tactical review with players in realistic first-person or panoramic scenarios [7,22,36]. This suggests that VR is not only a training interface but also a teaching interface, a shared space where players and staff can review decisions, constraints, and alternatives without the physical and schedule demands of a full training session. Simultaneously, immersive systems capture sensitive data, such as gaze patterns, decision latency, biomechanical signatures, and, in some cases, neurocognitive indicators. This raises ethical questions about storage, consent, access, and secondary use of these data, especially when working with minors, as well as practical questions about cost, portability, space requirements, cybersickness risk, and staffing burden for setup and supervision [41,42]. Future VR-in-soccer studies should therefore (i) include multiple roles in the soccer ecosystem, including players, referees, coaches, analysts, and rehabilitation staff, rather than focusing only on outfield players; (ii) report feasibility metrics such as cost of hardware, typical setup time, frequency and severity of cybersickness, and staff training load; and (iii) explicitly address governance of behavioral, biometric, and neurocognitive data in youth and school settings [42,43]. This reporting will be critical for educational stakeholders, who must evaluate not only whether VR “works,” but also whether it is safe, affordable, and responsible to deploy.

#### 4.5.7. Toward Responsible, Scalable Adoption of VR in Student Soccer

Ultimately, progress in this field will depend not only on technical sophistication but also on practical viability and ethical governance. For VR to become part of routine soccer education rather than an occasional demonstration, systems must be affordable, portable, and simple enough to deploy in schools and academies; they must minimize cybersickness and staff burden; and they must include clear, developmentally appropriate policies for collecting, storing, and using behavioral, biometric, and neurocognitive data from minors.

At the same time, researchers must move beyond short-term proof-of-concept findings and determine whether any durable benefits transfer to real match behavior, whether VR can contribute to injury-risk management or return-to-play support, and whether these applications can be implemented without introducing additional safety, accessibility, ethical, or operational concerns. The agenda outlined above—standardized reporting, longitudinal and transfer-focused designs, adaptive individualized workloads, inclusion of diverse soccer roles, and explicit attention to feasibility, ethics, and data stewardship—describes a research and implementation agenda for determining whether VR can become a sustainable and developmentally responsible component of student-soccer education.

## 5. Conclusions

Evidence for VR in student and youth soccer is promising but remains preliminary. Controlled intervention studies suggest possible short-term improvements in selected tactical, perceptual-cognitive, technical, sensorimotor, and engagement outcomes. However, these findings should be interpreted cautiously because the evidence base comprises a small number of heterogeneous studies with varied populations, intervention designs, comparison conditions, and outcome measures. Cross-sectional and profiling studies indicate that some VR tasks may distinguish players by expertise, age, or competitive level, supporting discriminative, construct, or predictive validity; however, these studies do not demonstrate that VR training causes performance improvement.

This review provides an integrated synthesis of VR sensing configurations and evidence purposes across school, academy, university, and selected indirect adult soccer contexts. Head-mounted displays, 360° projection systems, eye tracking, inertial sensors, electromyography, and electroencephalography were used across training, assessment, rehabilitation-related, and educational applications. Nevertheless, technical reporting was frequently incomplete, and no included study directly compared alternative sensing configurations within the same participant sample. Consequently, no conclusion can be made regarding the superiority of one display, tracking, or sensor architecture.

Rehabilitation and injury-related evidence remains especially limited and is partly derived from recreational-adult or clinical populations rather than directly eligible student or youth samples. Findings concerning balance, postural control, heading accuracy, neuromuscular function, or confidence should therefore be interpreted as changes in performance or surrogate outcomes rather than evidence of reduced injury incidence, lower reinjury risk, or safer return to play. Engagement findings indicate possible educational value, particularly for motivation, attention, enjoyment, and participation; however, usability, accessibility, presence, cognitive workload, cybersickness, physical comfort, and sustained user experience were not systematically assessed.

Overall confidence in the evidence is limited by methodological heterogeneity; small and predominantly (though not exclusively) male samples; mixed student, youth, adult, and clinical populations; inconsistent comparison conditions; incomplete technical reporting; and short follow-up periods. Most improvements were measured inside VR, in laboratory assessments, controlled technical tests, or small-sided games. Evidence that these gains persist over time or transfer to full competitive-match performance remains weak. Therefore, in-VR or short-term laboratory improvements should not be interpreted as established real-world soccer-performance benefits.

Accordingly, VR should currently be regarded as a potentially useful complementary tool for soccer training, assessment, rehabilitation-related practice, and education rather than as an established replacement for conventional methods. Larger and adequately powered controlled studies using clearly defined student and youth populations, standardized comparison conditions, delayed retention testing, and ecologically valid full-match outcomes are required before firm conclusions can be made regarding durable performance enhancement, injury reduction, return-to-play effectiveness, or broad educational adoption.

## Figures and Tables

**Table 1:** Study and population characteristics.

Country	Population Classification	Participants	Soccer Context	Study Design	Comparator	Evidence Purpose
United States [14]	Collegiate student-athletes	*n* = 130; mean age ≈ 20.5 years; men and women	NCAA Division II soccer	Quasi-experimental controlled trial	Usual strength and conditioning	Training effectiveness; injury-related surrogate outcomes
Germany [6]	Youth and young-adult goalkeepers across expertise levels	*n* = 35; 12 experts, 10 intermediate-level players, and 13 novices	Elite, regional-league, and amateur goalkeeping	Cross-sectional laboratory assessment	Expert, intermediate, and novice groups	Assessment/discriminative validity
United Kingdom [18]	Professional, academy, and novice participants	Professionals: *n* = 17, mean age 28.4; academy: *n* = 17, mean age 14.5; novices: *n* = 17, mean age 21.5 years	Soccer-specific VR assessment	Cross-sectional comparative study	Professional vs. academy vs. novice groups	Assessment/construct validity
South Korea [13]	Elementary-school students	*n* = 113; Grade 5; mean age ≈ 11 years	School physical education	Quasi-experimental two-group study	Traditional physical-education classes	Engagement and educational effectiveness
Germany [19]	Male academy players across age and performance levels	*n* = 292; mean age 14.8 ± 2.8 years; U12–U23	Elite and sub-elite academies from two professional clubs	Cross-sectional profiling study	Elite vs. sub-elite players across age groups	Assessment/discriminative validity
Brazil [20]	National-level male youth players	*n* = 26; age 15.4 ± 0.3 years	Competitive youth soccer	Randomized parallel-group trial	Video-based tactical instruction	Tactical and perceptual-cognitive training effectiveness
Germany [21]	Youth soccer players	*n* = 42; age 11–13 years; mean ≈ 12.3 years	Four recreational youth teams	Three-group intervention; randomization not specified	Perceptual-cognitive training vs. pseudo-video active control vs. usual practice	Tactical and perceptual-cognitive training effectiveness
Germany [4]	General adult participants	*n* = 37; age 24.89 ± 2.99 years; 25 men and 12 women	Simulated soccer cognitive tasks	Within-subject user study	VR vs. non-VR versions of the same tasks	Engagement, usability, and feasibility
Germany [22]	Youth academy and regional-league players	*n* = 48 male U17/U19 players; mean age ≈ 17.5 years	Youth academy and regional soccer	Cross-sectional assessment with three-year follow-up	Academy vs. regional league; U17 vs. U19	Assessment and predictive validity
United Kingdom [15]	Recreational adult soccer players	VR: *n* = 18, mean age ≈ 24.2; control: *n* = 18, mean age ≈ 28.7 years	Home-based heading practice	Randomized controlled trial	No heading practice for 7–10 days	Technical training effectiveness; indirect injury-related application
Iran [16]	Male soccer players with chronic ankle instability	*n* = 32; age 18–40 years	University- and club-level recreational soccer	Randomized single-blind controlled trial	Traditional balance exercises	Rehabilitation effectiveness; indirect adult evidence
France [23]	Elite youth academy players	*n* ≈ 16	Elite youth football academy	Pilot experimental intervention	Routine training or control condition insufficiently described	Training effectiveness and preliminary field transfer
Italy [5]	Semi-elite youth academy players	*n* = 30 male U16/U17 players; age 15.6 ± 0.5 years	Competitive youth academy soccer	Randomized controlled trial; eight-week mixed 2 × 2 design	Standard soccer training	Perceptual-cognitive training effectiveness
Turkiye [24]	Male youth football players	*n* = 22; U16–U17	Youth football training	Randomized controlled study; seven-week intervention	Traditional football training	Tactical and perceptual-cognitive training effectiveness
Japan [25]	Participants completing heading training	*n* = 64; allocated to five groups	Soccer heading-skill development	Randomized controlled study; eight-week intervention	Ball, lightweight ball, combined, and control/comparison conditions	Technical training effectiveness; controlled-rehearsal application
France [26]	Soccer players across skill levels	*n* = 24; 12 high-skilled and 12 less-skilled players	Virtual soccer perceptual-cognitive assessment	Experimental within-task comparative assessment	Conditions with and without ball-reception demands	Assessment/construct validity
Malaysia [27]	Youth football players	*n* = 60; mean age 21.08 ± 1.49 years	Youth football skills and imagery training	Randomized three-group intervention; 12 weeks	VR imagery vs. conventional imagery script vs. control	Technical training effectiveness and engagement
Indonesia [28]	Young male soccer players	*n* = 20; age 11–13 years; VR *n* = 10; control *n* = 10	Youth soccer training center	Randomized controlled pretest–posttest trial; 8 weeks; 3 sessions/week	Conventional youth soccer training	Physical and sensorimotor training effectiveness
Indonesia [29]	Male beginner-level young soccer athletes	*n* = 50; age 17–19; VR + SSG *n* = 25; SSG-only *n* = 25	Young soccer training	Randomized true experimental pretest–midtest–posttest design	Small-sided-game training only	Technical training effectiveness; teamwork and engagement/motivation
Jordan [30]	Adolescent female academy football players	*n* = 80; 14–18 years; mean age 15.8 ± 1.2 years; 20/group	Football training academy (AL WEFAC)	Six-month quasi-experimental comparative study; four coaching groups	Traditional coaching; self-paced online modules; blended coaching; VR-assisted coaching	Physical-training effectiveness; psychological/engagement outcomes

**Note:** Assessment studies evaluated whether VR tasks distinguished expertise, competitive level, or future progression and were not interpreted as evidence that VR training improved performance. Studies involving recreational adults or clinical adult populations were treated as indirect evidence and were not used to make direct conclusions about student or youth training effectiveness.

**Table 2:** Outcomes and quantitative findings.

Study	Outcome Domain	Measurement Instrument/Outcome	Assessment Setting	Follow-Up	Quantitative Result Available in Current Extraction	Effect Estimate/95% CI	Evidence Interpretation
[14]	Sensorimotor and neuromuscular control	In-VR task accuracy, stability and coordination; balance, oculomotor and cervical-function assessments; InStat performance index	In-VR, laboratory/off-VR and match statistics	Post-intervention plus 10-week post-training follow-up in the experimental cohort.	Significant between-group training effects were observed for 7/9 VR exercises: smooth pursuits, coefficient = **74.18**, *p* < 0.0001; saccades = **95.78**, *p* < 0.0001; near-point convergence = **38.02**, *p* = 0.005; peripheral vision = **43.98**, *p* < 0.0001; visual figure = **127.73**, *p* < 0.0001; joint-position target = **41.93**, *p* = 0.0001; neuromotor maze = **41.96**, *p* = 0.0001. Clinical improvements included CCFT, *p* < 0.0001; deep-neck-flexor endurance, *p* = 0.009; and overall balance, *p* = 0.0001. Smooth-pursuit VR score was associated with InStat match performance, *p* = 0.022. No protective effect on injury incidence or overall match-performance difference was demonstrated. A 10-week post-training follow-up was also performed.	Examples of reported lower 97.5% CI bounds: smooth pursuits **45.44**, saccades **68.92**, peripheral vision **27.01**, visual figure **102.74**. No standardized overall effect size reported.	Strong evidence of improvement in selected **trained and near-transfer sensorimotor measures**, but no evidence of reduced injury incidence or general improvement in match performance.
[6]	Gaze behavior and expertise classification	Fixation duration, saccade amplitude, pursuit dispersion, velocity and acceleration; support-vector-machine classification	Laboratory assessment	Single-session assessment	The best three-class SVM model achieved median classification accuracy of **78.20%**, compared with chance level 33.33%; median recall was **76.18%**. Other model accuracies were 75.08% and 73.95%.	Classification accuracy **78.2%**; 95% CI not reported.	Supports **discriminative validity** of eye-movement features for expertise classification; no training effect was tested.
[18]	Tactical assessment and construct validity	Rondo Scan, Color Combo, Shoulder Sums and Pressure Pass scores; reaction time, passing accuracy and Rezzil Index	In-VR	Single-session assessment	Overall MANOVA: Pillai’s Trace = **0.91**, F (18,82) = **3.82**, *p* < 0.001, ηp^2^ = **0.46**. Rezzil Index: novices **51.75**, academy **58.08**, professionals **69.03**; F (2,48) = **28.23**, *p* < 0.001. Professional players also outperformed other groups on Rondo Scan, Shoulder Sums, Pressure Pass and several process measures.	Overall MANOVA ηp^2^ = **0.46**. Rezzil Index pairwise Cohen’s d: novice–academy **0.98**; academy–professional approximately large; novice–professional very large. Authors reported differentiation probabilities of **76%, 85%, and 97%** for novice–academy, academy–professional, and novice–professional comparisons, respectively.	Strong construct/discriminative-validity evidence for the overall Rezzil Index; does not establish training effectiveness or real-world transfer.
[13]	Engagement and educational outcomes	Participation, interaction frequency, class-flow, motivation, attention, enjoyment and task-feedback scores	School physical-education setting; self-report and system records	Post-intervention only; retention not reported	For class attitude, significant Time × Group effects were found for **confidence**, F (1,109) = **4.369**, *p* = **0.039**, and **concentration**, F (1,109) = **4.479**, *p* = **0.037**; interest was not significant, F (1,109) = 0.170, *p* = 0.681. The VR group also showed significant advantages for selected class-flow dimensions	Standardized effect sizes were not reported in the accessible article tables; 95% CIs not reported.	Provides school-based evidence for selected **confidence, concentration, and flow outcomes**, but not athletic-performance effectiveness or long-term educational benefit.
[19]	Multiple-object tracking and attention	Percentage of correctly identified targets over 30 trials; Spearman correlations; 8 × 2 ANOVA	In-VR assessment	Single-session assessment	360-MOT performance correlated with age, **rs = 0.33, *p* < 0.001**, and soccer experience, **rs = 0.31, *p* < 0.001**. Older age groups performed better than U12, and elite players performed better than sub-elite players, particularly in U19/U23 groups.	Correlation effects: **rs = 0.33** for age and **rs = 0.31** for soccer experience. Exact ANOVA effect-size estimates were not reported in the extracted study results.	Supports age- and expertise-related **discriminative validity** of the 360-MOT assessment; no training effect was tested.
[20]	Tactical decision-making and visual search	Small-sided-game decision accuracy; fixation count and duration; Stroop test	Small-sided game and laboratory assessment	Immediate post-intervention; retention not reported	Significant Group × Time interactions were reported for **decision-making performance, *p* < 0.01**, and **visual-search behavior, *p* < 0.01**. Both groups improved, but improvements were significantly greater in VR, *p* < 0.01. Inhibitory control improved in both groups, *p* < 0.01, but the Group × Time interaction was not significant, *p* > 0.05.	Not reported and not calculable from the published data (NR–NC).	Controlled evidence of short-term improvement in decision-making and visual-search behavior compared with video training; full competitive-match transfer and retention were not tested.
[21]	Attention, tracking, passing and decision-making	360° MOT accuracy; visuospatial reaction time; Footbonaut passing accuracy and execution time; small-sided-game scoring	In-VR, laboratory and small-sided game	Immediate post-intervention; long-term retention not reported	At baseline, 360-MOT correlated with 360° passing accuracy, **r = 0.31, *p* = 0.045**, and defensive small-sided-game performance, **r = 0.35, *p* = 0.031**. After five weeks, the training group showed significant **task-specific 360-MOT improvement**, but **no significant transfer effects** to other perceptual-cognitive or soccer-specific performance measures.	Correlations: **r = 0.31** and **r = 0.35**. Exact standardized training-effect estimate was not available in the accessible publisher text.	Evidence supports **task-specific training effects**, not confirmed transfer to soccer-specific performance. This is more accurate than “greater improvement in tactical performance.”
[4]	Selected usability and user-experience outcomes	Five-point effort-expectancy and hedonic-motivation scales; VR versus non-VR preference or experience ratings where reported; paired t tests	VR and non-VR within-subject comparison	Single-session assessment	Effort expectancy: non-VR **4.12 ± 0.24** vs. VR **4.00 ± 0.19**; t (37) = **1.31**, *p* = **0.20**. Hedonic motivation: non-VR **3.69 ± 0.20** vs. VR **3.94 ± 0.21**; t (37) = **−2.44**, *p* = **0.03**.	Cohen’s d = **0.536** for effort expectancy and **−1.151** for hedonic motivation, as reported by the authors.	VR increased hedonic motivation without significantly increasing perceived effort. This is **limited usability/UX evidence**, not soccer-training effectiveness.
[22]	Decision-making and predictive validity	Decision accuracy; ROC analysis; odds ratios; ANOVA; immersion ratings and interviews	In-VR assessment plus prospective follow-up	Three-year follow-up	Youth-academy players: **69.06 ± 9.08%** decision accuracy vs. regional-level players **58.80 ± 8.55%**; performance-level effect F (1,44) = **18.07**, *p* < 0.001, η^2^ = **0.29**. U19 vs. U17 effect: F (1,44) = **7.04**, *p* < 0.01, η^2^ = **0.14**. Three-year follow-up: future higher-level players **72.22 ± 7.52%** vs. lower-level players **65.32 ± 9.66%**, t (22) = **−1.97**, *p* < 0.05. AUC = **0.71**; optimal cut-off 71%; OR = **6.00**	Diagnostic η^2^ = **0.29** for performance level and **0.14** for age. Prospective difference d = **0.80**; AUC = **0.71**; OR = **6.00**; lower 90% CI for AUC = **0.52**.	Strong discriminative and preliminary prognostic-validity evidence. It was **not a training intervention**, so do not interpret these results as VR-induced improvement.
[15]	Heading accuracy and confidence	Goal-scoring rate, shot-placement accuracy and self-rated heading ability/confidence	Controlled field/video-coded assessment and self-report	After 7–10 days of practice	Goals scored: Group × Time F (1,34) = **5.42**, *p* = **0.026**, ηp^2^ = **0.137**; VR group improved pre–post, *p* = **0.042**, control did not, *p* = 0.558. Shot accuracy interaction was not significant: F (1,33) = **2.78**, *p* = **0.105**, ηp^2^ = **0.078**. VR confidence increased: Z = **3.39**, *p* < 0.001; self-efficacy: Z = **2.85**, *p* = 0.004.	Goals interaction ηp^2^ = **0.137**; shot-accuracy interaction ηp^2^ = **0.078**.	VR heading practice improved number of goals and self-reported confidence/self-efficacy but not shot accuracy. No head-impact, injury-incidence, or reinjury outcome was assessed.
[16]	Rehabilitation and postural control	Normalized EMG, muscle-onset latency, Y-Balance Test and Cumberland Ankle Instability Tool	Laboratory rehabilitation assessment	Immediate post-intervention; longer follow-up not reported	VR group: SOL activity during CPA2 decreased, ***p* = 0.033**; BF activated earlier, ***p* = 0.043**. Both groups improved perceived ankle instability (VR ***p* = 0.002**; balance training ***p* < 0.001**) and Y-Balance outcomes (VR ***p* < 0.021**; balance training ***p* < 0.033**). Between groups, balance training showed greater GM amplitude changes during APA/CPA1 (***p* = 0.050** and ***p* = 0.012**).	Exact standardized between-group effects were not reported in the abstract; 95% CIs were not available there.	Both VR and conventional balance training improved selected rehabilitation outcomes. **No clear overall superiority of VR**, and reinjury/RTP outcomes were not measured.
[23]	Scanning and passing behavior	Pre-reception scanning frequency and passing success	Small-sided game/controlled field assessment	Immediate post-intervention; retention not assessed	Pre-reception scanning: VR **0.073 ± 0.040 → 0.109 ± 0.055 scans/s (+49.3%)**; control **0.070 ± 0.032 → 0.069 ± 0.035 (−1.4%)**. Group × Time F = **2.56**, *p* = **0.132**. Passing success: F = **0.023**, *p* = **0.882**.	Scanning η^2^ = **0.155** (large); passing η^2^ = **0.002** (trivial); 95% CIs not reported.	Descriptive scanning increased with a large effect estimate, but **between-group effect was nonsignificant**. Evidence of field transfer remains inconclusive.
[5]	Cognitive performance and neural preparation	Go/No-Go reaction time and accuracy; EEG prefrontal negativity and readiness potential; mixed ANOVA	Laboratory and neurophysiological assessment	Immediate post-intervention; long-term retention not reported	Reaction time showed Group × Time F (1,28) = **12.3**, *p* = **0.002**, ηp^2^ = **0.305**. VR group improved from **473 ± 49 ms to 436 ± 37 ms**, *p* = **0.003**; control remained similar, **479 ± 44 to 477 ± 31 ms**, *p* = 0.755. Prefrontal negativity increased by approximately **40%** in the VR group, and premotor BP was greater after training.	RT interaction ηp^2^ = **0.305**; Group main effect ηp^2^ = **0.183**. 95% confidence intervals for ERP changes were not reported.	Provides controlled evidence of short-term cognitive and anticipatory neurophysiological change; no full-match transfer or long-term retention measure.
[24]	Scanning and passing performance	Scanning before ball reception, during ball control, and off the ball; passing-performance categories	Small-sided-game video analysis	Immediate post-intervention after 7 weeks; delayed retention not reported	Between-group scanning: before reception ***p* < 0.001, q = 0.010, ES = 0.83**; during control ***p* = 0.006, q = 0.042, ES = 0.58**; off-ball ***p* < 0.001, q = 0.010, ES = 0.78**. Within VR group, all scanning domains improved, ***p* = 0.003, ES = 0.89**. One-touch short-successful passes increased **38%, *p* = 0.006, ES = 0.83**; multi-touch effects were nonsignificant.	Between-group ES = **0.58–0.83**; within-group scanning ES = **0.89**; one-touch short-pass ES = **0.83**.	Controlled evidence of improved scanning. Passing effects were selective and should not be generalized to overall passing performance.
[25]	Heading accuracy and strength	Heading accuracy and ball-flight distance	Controlled heading-performance test	Immediate post-intervention after eight weeks	After eight weeks, **VR, soccer-ball, and combined VR + ball groups significantly improved heading accuracy**; lightweight-ball and control groups did not. Ball-flight distance increased only in the soccer-ball and combined groups; VR alone did not significantly improve flight distance. Exact F/p statistics are not exposed in the accessible publisher abstract.	Not reported and not calculable from the published data (NR–NC).	VR appears useful for heading accuracy, whereas physical ball practice remained necessary for improving ball-flight distance/strength. VR should therefore be considered complementary rather than a replacement for physical heading practice.
[26]	Visual tracking and attention allocation	Multiple-player tracking performance and global displacement across ball-reception conditions	CAVE/immersive laboratory assessment	Single session assessment	Visual tracking: condition effect F (2,44) = **30.64**, *p* < 0.001, ηp^2^ = **0.58**; no skill-group main effect F (1,22) = 0.39, *p* = 0.540, ηp^2^ = 0.02; no interaction F (2,44) = 0.96, *p* = 0.392, ηp^2^ = 0.04. ST-Track **2.61 ± 0.28** vs. TrackNoPass **2.25 ± 0.37**, t (23) = −6.08, *p* < 0.001, d = 1.24; vs. dual-task TrackPass **2.16 ± 0.30**, t (23) = −7.93, *p* < 0.001, d = 1.62. Reception success was unrelated to condition or skill group.	Condition ηp^2^ = **0.58**; pairwise d = **1.24** and **1.62**.	VR assessment was sensitive to **task-load effects**, but not to player skill level in this sample. This is assessment evidence, not training-effectiveness evidence.
[27]	Kicking performance and motivation	Mor–Christian Football Kicking Test and Sports Motivation Scale-6	Field/technical test and self-report	Immediate post-intervention after 12 weeks	After 12 weeks, kicking performance differed significantly across groups, **F (2,57) = 5.53, *p* = 0.006**. Post-intervention kicking means were approximately **68.0** for VR imagery, **59.0** for conventional imagery, and **59.7** for control. The VR imagery group also showed the strongest increase in motivation; exact full ANOVA statistics for all motivation subscales were not reported in sufficient detail to extract reliably.	Standardized effect size and 95% CI were not reported in the accessible chapter results.	Preliminary evidence that VR imagery may improve kicking performance and motivation, but no competitive-match transfer or retention assessment was performed.
[28]	Balance and agility; physical/sensorimotor training	Stork Static Balance Test; T-Test agility assessment	Youth soccer training/testing environment	Immediate post-intervention after **8 weeks**; no delayed retention reported	Significant Time × Group interactions favored VR training for balance (**F = 124.35, *p* < 0.001**) and agility (**F = 26.21, *p* < 0.001**).	VR-group within-group change: balance Δ = 4.87 s, d = 1.07; agility Δ = 0.83 s, d = 2.70. Control-group changes were Δ = 1.37 s, d = 0.54 and Δ = 0.31 s, d = 1.05, respectively; 95% CIs not reported.	Controlled evidence suggests substantial short-term improvement in balance and agility following VR-supported multimodal training. However, delayed retention and transfer to competitive-match performance were not established.
[29]	Technical performance; teamwork; engagement/motivation	Passing accuracy; shooting accuracy; dribbling performance; teamwork measures; intention to continue exercising	3-D VR soccer training followed by small-sided-game/field-based assessment	Pretest, midtest, and posttest across the intervention period; delayed retention not reported	Significant Training Condition × Time interactions favored the 3-DVR + SSG condition for intention to continue exercising (F = 15.83, *p* < 0.001, ηp^2^ = 0.152), offensive teamwork (F = 13.47, *p* < 0.001, ηp^2^ = 0.133), defensive teamwork (F = 15.23, *p* < 0.001, ηp^2^ = 0.145), communication (F = 11.47, *p* = 0.002, ηp^2^ = 0.126), passing accuracy (F = 11.29, *p* = 0.003, ηp^2^ = 0.114), shooting accuracy (F = 10.74, *p* < 0.001, ηp^2^ = 0.109), and dribbling speed (F = 9.86, *p* = 0.007, ηp^2^ = 0.101).	ηp^2^ = 0.101–0.152 for Training Condition × Time interactions; 95% CIs not reported.	Combined 3-D VR + small-sided-game training improved selected technical and psychosocial outcomes relative to small-sided-game training alone. Because VR was part of a combined intervention, the findings should not be attributed to VR alone.
[30]	Physical performance; psychological and engagement outcomes	Illinois Agility Test; Yo-Yo IR1; 30 m sprint; Sport Confidence Inventory; Group Environment Questionnaire; Perceived Stress Scale (PSS-10)	Football academy training environment; physical-performance testing and questionnaire-based psychological assessment	Assessments at baseline, 3 months, and 6 months	The study compared blended coaching, traditional coaching, VR-assisted coaching, and self-paced online training. Overall multidimensional evaluation favored blended coaching, followed by VR-assisted coaching; PROMETHEE analysis ranked Blended Coaching first (φ = +1.50), followed by VR-Assisted Coaching (φ = +0.60), Traditional Coaching (φ = −0.90), and Self-paced Online Modules (φ = −1.20).	PROMETHEE net-flow score for VR-assisted coaching: φ = +0.60; conventional standardized effect size and 95% CI not reported/calculable.	Provides potentially relevant evidence that VR-assisted coaching may support physical and psychological development in adolescent female academy footballers. However, results from a multidimensional ranking approach should not be interpreted as a conventional standardized intervention effect.

**Note:** Quantitative results were extracted directly from the original included articles. Author-reported group means, standard deviations, percentage changes, test statistics, exact *p*-values, effect sizes, confidence intervals, correlations, odds ratios, and classification-performance measures were reported where available. No numerical value was estimated when the published article did not provide sufficient information. “NR–NC” indicates that the relevant effect estimate or precision measure was not reported and could not be calculated from the published data. Where effect sizes were reported by the original authors, the original effect-size metric and interpretation were retained. Bold values indicate statistically significant results.

## Data Availability

No new data were created or analyzed in this study. Data sharing is not applicable to this article.

## Supplementary Materials

The following supporting information can be downloaded at: https://www.mdpi.com/article/10.3390/s26175381/s1.
