# Supplementary material for: Virtual Reality Sensing Technologies in Student Soccer Training: A Systematic Review of Tracking Systems and Perceptual-Cognitive, Physical, Tactical, and Educational Outcomes
Source: Sensors (Basel). 2026 Aug 26;26(17):5381. doi: 10.3390/s26175381 (PMC13568064; doi:10.3390/s26175381)
Supplement: Supplementary file 1 [file sensors-26-05381-s001.zip › sensors-4441648-supplementary.pdf]

PRISMA 2020 Checklist
Virtual Reality Sensing Technologies in Student Soccer Training: A Systematic Review of Tracking Systems and Perceptual-Cognitive, Physical, Tactical, and Educational Outcomes

OSF Registration: https://doi.org/10.17605/OSF.IO/WX2G5

#	Item	Checklist item	Location in manuscript	Reported
**TITLE**				
1	**Title**	Identify the report as a systematic review.	Title page	✓
**ABSTRACT**				
2	**Abstract**	See the PRISMA 2020 for Abstracts checklist. Includes background, objectives, eligibility criteria, information sources, risk of bias, synthesis methods, results, limitations, and conclusions.	Abstract (p.1)	✓
**INTRODUCTION**				
3	**Rationale**	Describe the rationale for the review in the context of existing knowledge.	Section 1	✓
4	**Objectives**	Provide an explicit statement of the objective(s) or question(s) the review addresses.	Section 1	✓
**METHODS**				
5	**Eligibility criteria**	Specify the inclusion and exclusion criteria for the review and how studies were grouped for the syntheses.	Section 2.3	✓
6	**Information sources**	Specify all databases, registers, websites, organizations, reference lists, and other sources searched or consulted to identify studies. Specify the date when each source was last searched or consulted.	Section 2.2; **Supplementary Table S1**	✓*
7	**Search strategy**	Present the full search strategies for all databases and registers, including any filters applied.	Section 2.2; **Supplementary Table S1**	✓*
8	**Selection process**	Specify the methods used to decide whether a study met the inclusion criteria of the review, including how many reviewers screened each record and each report retrieved,	Section 2.4	✓

#	Item	Checklist item	Location in manuscript	Reported
		whether they worked independently, and if applicable, details of automation tools used.		
9	**Data collection process**	Specify the methods used to collect data from reports, including how many reviewers collected data from each report, whether they worked independently, any processes for obtaining or confirming data from study investigators, and if applicable, details of automation tools used.	Section 2.4	✓
10	**Data items**	List and define all outcomes for which data were sought. Specify whether all results that were compatible with each outcome domain in each study were sought, and if not, the methods used to decide which results to collect.	Section 2.4; Tables 1–2; Supplementary Table S3	✓
11	**Study risk of bias assessment**	Specify the methods used to assess risk of bias in the included studies, including details of the tool(s) used, how many reviewers assessed each study and whether they worked independently, and if applicable, details of automation tools used.	Section 2.5	✓
12	**Effect measures**	Specify for each outcome the effect measure(s) (e.g. risk ratio, mean difference) used in the synthesis or presentation of results.	Section 2.4; Table 2	✓§
13	**Synthesis methods**	Describe the processes used to decide which studies were eligible for each synthesis, how data were prepared for synthesis, any methods used to tabulate or visually display results of individual studies and syntheses, and any methods used to synthesize results.	Sections 2.5; 3	✓
14	**Reporting bias assessment**	Describe any methods used to assess risk of bias due to missing results in a synthesis (arising from reporting biases).	Section 4.3	✓
15	**Certainty assessment**	Describe any method used to assess certainty (or confidence) in the body of evidence for an outcome.	Not applicable	N/A¶
**RESULTS**				
16	**Study selection**	Describe the results of the search and selection process, including the number of records identified in the search and	Section 2.4; Figure 2	✓

#	Item	Checklist item	Location in manuscript	Reported
		number of records excluded at each stage of selection, with reasons for exclusions. Ideally, present a flow diagram.		
17	**Study characteristics**	Each of the site includes study and present its characteristics.	Table 1; Section 3	✓
18	**Risk of bias in studies**	Present assessments of risk of bias for each included study.	Section 3.2; Supplementary Tables S2.1–S2.4	✓
19	**Results of individual studies**	For all outcomes, present, for each study: (a) summary statistics for each group (where appropriate) and (b) an effect estimates and its precision, ideally using structured tables or plots.	Table 2; Sections 3.3–3.7	✓†
20	**Results of syntheses**	For each synthesis, briefly summarize the characteristics and risk of bias among contributing studies. Present results of all statistical syntheses conducted. If meta-analysis was done, present for each the summary estimate and its precision.	Sections 3.7; 4.1	✓†
21	**Reporting biases**	Present assessments of risk of bias due to missing results (arising from reporting biases) for each synthesis assessed.	Section 4.3	✓
22	**Certainty of evidence**	Present assessments of certainty (or confidence) in the body of evidence for each outcome assessed.	Not applicable	N/A¶
**DISCUSSION**				
23	**Discussion**	Provide a general interpretation of the results in the context of other evidence. Discuss any limitations of the evidence included in the review. Discuss any limitations of the review processes used. Discuss implications of the results for practice, policy, and future research.	Sections 4.1–4.5	✓
**OTHER INFORMATION**				
24	**Registration and protocol**	Provide registration information for the review, including register name and registration number, or state that the review was not registered. Indicate where the review	Abstract; Section 2.1	✓‡

#	Item	Checklist item	Location in manuscript	Reported
		protocol can be accessed, or state that a protocol was not prepared.		
**25**	**Support**	Describe sources of financial or other support for the review, and the role of the funders or sponsors in the review.	Funding section	✓
**26**	**Competing interests**	Declare any competing interests of review authors.	Declaration section	✓
**27**	**Availability of data, code, and other materials**	Report which of the following are publicly available and where they can be found: template data collection forms; data extracted from included studies; data used for all analyses; analytic code; any other materials used in the review.	Data availability; OSF	✓

Notes:

* Items 6–7 (Information sources; Search strategy): All information sources are identified and reconstructed search strategies are reported. The exact original execution date for each database and the exact originally saved search syntax from the historical (pre-31 March 2026) search were not retained and could not be reconstructed. To address this reproducibility limitation, all five database searches were rerun on 16 August 2026 using the identical reported search strategies; the exact execution date and database-specific retrieval counts for this update search are fully documented in Supplementary Table S1.

† Items 19–20 (Results of individual studies; Results of syntheses): Results of individual studies are presented in Table 2 and synthesized narratively in Sections 3.3–3.7. No pooled statistical estimates were computed because of substantial methodological heterogeneity.

‡ Item 24 (Registration): This review was not prospectively registered in PROSPERO prior to data collection, which is acknowledged as a methodological limitation in Section 4.3. A retrospective registration was completed on the Open Science Framework (OSF: https://doi.org/10.17605/OSF.IO/WX2G5) to enhance transparency.

§ Item 12 (Effect measures): Because substantial methodological heterogeneity precluded use of a single common effect measure or meta-analysis, author-reported effect measures were retained in their original metrics, including standardized effect sizes, η^2/partial η^2, correlations, odds ratios, classification accuracy, and other study-specific quantitative estimates where available. These are presented in Table 2.

¶ Items 15 and 22 (Certainty assessment; Certainty of evidence): A formal certainty-of-evidence framework such as GRADE was not applied because of substantial heterogeneity in study designs, populations, interventions, outcome domains, and effect measures. Risk-of-bias and methodological-quality assessments were instead used to inform the cautious narrative interpretation of the evidence.

Reference: Page MJ, et al. The PRISMA 2020 statement: an updated guideline for reporting systematic reviews. BMJ 2021;372:n71. doi: 10.1136/bmj.n71. For more information, visit: http://www.prisma-statement.org/

Supplementary Table S1. Database-Specific Search Strategies, Including the 16 August 2026 Reproducibility/Update Search

Searches were originally conducted in PubMed/MEDLINE, Scopus, Web of Science Core Collection, SPORTDiscus, and ERIC for studies published between January 1, 2020, and March 31, 2026. The exact execution dates of the original searches and the separate database-level retrieval counts were not retained during the original review process. Consequently, these historical values could not be reconstructed reliably and are reported as "not retained." The database-specific strategies presented below were reconstructed from the core Boolean strategy described in the original review records and adapted to the syntax and search fields of each database. The original searches identified 230 records in total across the five databases. To address this reproducibility limitation, all five database searches were rerun on 16 August 2026 using the identical database-specific search strategies, field codes, and language restrictions, extending the publication window through 16 August 2026. The rerun retrieved 42 records from PubMed/MEDLINE, 96 from Scopus, 71 from Web of Science Core Collection, 34 from SPORTDiscus, and 15 from ERIC (258 records in total). These update-search counts represent the actual retrieval results obtained on 16 August 2026 and are not reconstructions of the unavailable historical counts. All 258 records retrieved during the update search were exported to EndNote, compared against the original consolidated reference library, and screened for duplication; the substantial majority corresponded to records already present in, or bibliographically equivalent to, records already screened during the original review. Records not previously assessed were screened using the same predefined eligibility criteria described in Section 2.3 of the main manuscript. This update and verification process identified three additional eligible studies: one study (Makhadmeh & Fayad, 2025) that fell within the original review period (January 2020–March 2026) but had not been captured by the original search, and two studies published after the original 31 March 2026 cutoff. These three studies are incorporated into Table 1 and Table 2 of the main manuscript as references [31]–[33], and into Supplementary Tables S2.1/S2.2, S2.4, and S3 below.

**Database/platform**	**Original search date**	**Search fields**	**Limits applied**	**Original records retrieved**
PubMed/MEDLINE	Not retained	MeSH; Title/Abstract	English; 1 Jan 2020–31 Mar 2026	Not retained
Scopus	Not retained	TITLE-ABS-KEY	English; 1 Jan 2020–31 Mar 2026	Not retained
Web of Science Core Collection	Not retained	Topic search, TS	English; 1 Jan 2020–31 Mar 2026	Not retained
SPORTDiscus via EBSCOhost	Not retained	TX search	English; 1 Jan 2020–31 Mar 2026	Not retained
ERIC via EBSCOhost	Not retained	TX search	English; 1 Jan 2020–31 Mar 2026	Not retained
Combined original total	—	—	—	230
Update/reproducibility search — 16 August 2026	16 Aug 2026	—	English; 1 Jan 2020–16 Aug 2026	—
PubMed/MEDLINE	16 Aug 2026	MeSH; Title/Abstract	English; 1 Jan 2020–16 Aug 2026	42

Scopus	16 Aug 2026	TITLE-ABS-KEY	English; 1 Jan 2020–16 Aug 2026	96
Web of Science Core Collection	16 Aug 2026	Topic search, TS	English; 1 Jan 2020–16 Aug 2026	71
SPORTDiscus via EBSCOhost	16 Aug 2026	TX search	English; 1 Jan 2020–16 Aug 2026	34
ERIC via EBSCOhost	16 Aug 2026	TX search	English; 1 Jan 2020–16 Aug 2026	15
Combined update-search total	16 Aug 2026	—	—	258

S1.1 PubMed/MEDLINE

The reconstructed PubMed/MEDLINE strategy was:

```
(
  "Virtual Reality"[MeSH Terms]
  OR "virtual reality"[Title/Abstract]
  OR "immersive virtual reality"[Title/Abstract]
  OR "immersive VR"[Title/Abstract]
  OR "360 video"[Title/Abstract]
  OR "360-degree video"[Title/Abstract]
)
AND
(
  "Soccer"[MeSH Terms]
  OR soccer[Title/Abstract]
  OR football[Title/Abstract]
)
AND
(
  student*[Title/Abstract]
  OR youth[Title/Abstract]
  OR adolescent*[Title/Abstract]
  OR academy[Title/Abstract]
  OR school*[Title/Abstract]
  OR universit*[Title/Abstract]
  OR collegiate[Title/Abstract]
```

```
)
AND
(
  "physical training"[Title/Abstract]
  OR "skill acquisition"[Title/Abstract]
  OR "tactical understanding"[Title/Abstract]
  OR "decision making"[Title/Abstract]
  OR "decision-making"[Title/Abstract]
  OR "injury prevention"[Title/Abstract]
  OR rehabilitation[Title/Abstract]
  OR motivation[Title/Abstract]
  OR engagement[Title/Abstract]
  OR cognitive[Title/Abstract]
  OR "perceptual-cognitive"[Title/Abstract]
)
```

Limits: English language; publication date January 1, 2020 to March 31, 2026.

S1.2 Scopus

```
TITLE-ABS-KEY(
  ("virtual reality" OR "immersive virtual reality" OR "immersive VR"
   OR "360 video" OR "360-degree video")
  AND
  (soccer OR football)
  AND
  (student* OR youth OR adolescent* OR academy OR school*
   OR universit* OR collegiate)
  AND
  ("physical training" OR "skill acquisition"
   OR "tactical understanding" OR "decision making"
   OR "decision-making" OR "injury prevention"
   OR rehabilitation OR motivation OR engagement
   OR cognitive OR "perceptual-cognitive")
)
```

Limits: English language; publication years 2020–2026. Records published after March 31, 2026 were excluded during eligibility screening.

S1.3 Web of Science Core Collection

```
TS=(
  ("virtual reality" OR "immersive virtual reality" OR "immersive VR"
   OR "360 video" OR "360-degree video")
  AND
  (soccer OR football)
```

```
  AND
  (student* OR youth OR adolescent* OR academy OR school*
   OR universit* OR collegiate)
  AND
  ("physical training" OR "skill acquisition"
   OR "tactical understanding" OR "decision making"
   OR "decision-making" OR "injury prevention"
   OR rehabilitation OR motivation OR engagement
   OR cognitive OR "perceptual-cognitive")
)
```

Limits: English language; publication years 2020–2026. Records published after March 31, 2026 were excluded during eligibility screening.

S1.4 SPORTDiscus via EBSCOhost

```
TX (
  ("virtual reality" OR "immersive virtual reality" OR "immersive VR"
   OR "360 video" OR "360-degree video")
  AND
  (soccer OR football)
  AND
  (student* OR youth OR adolescent* OR academy OR school*
   OR universit* OR collegiate)
  AND
  ("physical training" OR "skill acquisition"
   OR "tactical understanding" OR "decision making"
   OR "decision-making" OR "injury prevention"
   OR rehabilitation OR motivation OR engagement
   OR cognitive OR "perceptual-cognitive")
)
```

Limits: English; publication date January 1, 2020, to March 31, 2026.

S1.5 ERIC via EBSCOhost

```
TX (
  ("virtual reality" OR "immersive virtual reality" OR "immersive VR"
   OR "360 video" OR "360-degree video")
  AND
  (soccer OR football)
  AND
  (student* OR youth OR adolescent* OR academy OR school*
   OR universit* OR collegiate)
```

AND
("physical training" OR "skill acquisition"
OR "tactical understanding" OR "decision making"
OR "decision-making" OR "injury prevention"
OR rehabilitation OR motivation OR engagement
OR cognitive OR "perceptual-cognitive")
)

Limits: English; publication date January 1, 2020, to March 31, 2026.

S1.6 Record Consolidation and Duplicate Removal

Records retrieved from the five databases were imported into EndNote and consolidated into a single reference library. Potential duplicate records were identified using EndNote's automated duplicate-detection function based on matching bibliographic fields, including title, author, and publication year. All records flagged as possible duplicates were manually compared, with DOI and journal information consulted where available, before removal. During the original review, 42 duplicate records were removed from the 230 identified records, leaving 188 unique records for title and abstract screening. For the 16 August 2026 reproducibility/update search, the 258 records retrieved across the five databases (Supplementary Table S1) were similarly imported into EndNote and compared against the consolidated original library. Records that duplicated, or were bibliographically equivalent to, records already screened during the original review were excluded from further screening. The remaining previously unscreened records were assessed against the same predefined eligibility criteria (Section 2.3 of the main manuscript), which identified three additional eligible studies: Makhadmeh and Fayad (2025), which fell within the original review period but had not been captured previously, and two studies published after the original 31 March 2026 cutoff. These three studies are included as references [31]–[33] in the main manuscript and are reflected in Supplementary Tables S2.1/S2.2, S2.4, and S3 below.

Supplementary Table S2.1

RoB 2 assessments for randomized controlled studies

Abbreviations:

- **L:** Low risk
- **SC:** Some concerns
- **H:** High risk
- **NI:** No information

Study	Randomization process	Deviations from intended interventions	Missing outcome data	Outcome measurement	Selection of reported result	Provisional overall judgment
[25]	SC	SC	NI	SC	NI	Some concerns
[15]	SC	SC	NI	SC	NI	Some concerns
[16]	SC	SC	NI	L/SC	NI	Some concerns
[5]	SC	SC	NI	L/SC	NI	Some concerns
[27]	SC	SC	NI	SC	NI	Some concerns
[28]	SC	SC	NI	SC	NI	Some concerns
[30]	SC	SC	NI	SC	NI	Some concerns
[31]	SC	SC	NI	SC	NI	Some concerns
[32]	SC	SC	NI	SC	NI	Some concerns

Supplementary Table S2.2

ROBINS-I assessments for non-randomized intervention studies

Abbreviations:

- **L:** Low
- **M:** Moderate
- **S:** Serious
- **C:** Critical
- **NI:** No information

Study	Confounding	Participant selection	Intervention classification	Deviations from intended intervention	Missing data	Outcome measurement	Selective reporting	Provisional overall judgment
[14]	S	M	L	NI	NI	M	NI	Serious
[13]	S	M	L	NI	NI	S	NI	Serious
[23]	S	M	L	NI	NI	M	NI	Serious
[4]	M	M	L	M	NI	M	NI	Moderate
[26]	S	S	M	NI	NI	M	NI	Serious
[33]	S	M	L	NI	NI	M	NI	Serious

Supplementary Table S2.3

Downs–Black-based appraisal of cross-sectional and profiling studies

The table below therefore records provisional domain-level concerns.

Study	Reporting	External validity	Internal validity—bias	Internal validity—confounding	Power/sample adequacy	Provisional concern
[6]	Moderate	High concern	Moderate	High concern	High concern	Moderate–high
[22]	Moderate	Moderate concern	Moderate	High concern	Moderate concern	Moderate
[21]	Moderate	Moderate concern	Moderate	Moderate–high concern	Lower concern	Moderate
[24]	Moderate	Moderate concern	Moderate	Moderate–high concern	Moderate concern	Moderate
[29]	Moderate	High concern	Moderate	High concern	High concern	Moderate–high

Supplementary Table S2.4

Consolidated study-level summary

Study	Design	Tool	Provisional overall judgment	Primary concern
[14]	Quasi-experimental controlled	ROBINS-I	Serious	Confounding and unclear allocation
[6]	Cross-sectional profiling	Downs–Black adapted	Moderate–high concern	Small sample and expertise-group confounding
[22]	Cross-sectional comparative	Downs–Black adapted	Moderate concern	Age and expertise confounding
[13]	Quasi-experimental	ROBINS-I	Serious	Non-random assignment and subjective outcomes
[21]	Cross-sectional profiling	Downs–Black adapted	Moderate concern	Maturation and playing-level confounding
[25]	Randomized trial	RoB 2	Some concerns	Allocation concealment and missing-data reporting unclear
[23]	Three-group non-randomized/unclear intervention	ROBINS-I	Serious	Randomization not specified

[4]	Within-subject comparison	ROBINS-I/adapted appraisal	Moderate	Adult indirect sample and possible order effects
[24]	Cross-sectional plus follow-up	Downs–Black adapted	Moderate concern	Selection and developmental confounding
[15]	Randomized trial	RoB 2	Some concerns	No-practice control and small sample
[16]	Randomized single-blind trial	RoB 2	Some concerns	Blinding limitations and incomplete reporting
[26]	Pilot experimental	ROBINS-I	Serious	Small sample and unclear comparator/allocation
[5]	Randomized trial	RoB 2	Some concerns	Small sample and incomplete allocation/attrition reporting
[27]	Randomized trial	RoB 2	Some concerns	Small sample and insufficient methodological reporting
[28]	Randomized five-group study	RoB 2	Some concerns	Multiple comparisons and incomplete methodological reporting
[29]	Experimental cross-sectional assessment	Downs–Black adapted	Moderate–high concern	Small sample and no independent control
[30]	Randomized three-group intervention	RoB 2	Some concerns	Allocation concealment, attrition and blinding unclear
[31]	Randomized controlled trial	RoB 2	Some concerns	Small sample; allocation concealment and blinding details incompletely reported
[32]	Randomized controlled trial	RoB 2	Some concerns	Structural population pathway (school/academy/university) not explicitly confirmed in the source abstract; allocation procedure incompletely described
[33]	Quasi-experimental, four-arm comparison	ROBINS-I	Serious	Non-randomized allocation across coaching conditions and possible confounding by participant selection

Supplementary Table S3. VR Display, Tracking, Interaction, and Sensor Specifications.

Study	Display environment	Positional tracking	Interaction/response device	Auxiliary sensors or assessment systems	Analysis software/platform	Technical parameters
**[14]**	Immersive VR-based sensorimotor training system; exact HMD or display model not reported	Optical and inertial motion tracking reported; exact tracking architecture not reported	Task-specific VR interaction devices not reported	Oculomotor, balance, cervical-function, and sensorimotor assessment systems; exact device models not reported	Not reported	HMD model, degrees of freedom, latency, frame rate, field of view, sensor sampling rate, calibration, and tracking accuracy not reported
**[6]**	HTC Vive HMD presenting omnidirectional 4K soccer footage in an immersive spherical environment.	HMD-based head-orientation/VR tracking through the HTC Vive/SteamVR environment; detailed base-station configuration not reported.	Participant decision-response method used during stimulus trials; exact controller configuration not confirmed from the available report.	SMI high-speed eye tracker integrated with the HTC Vive; gaze sampling at 250 Hz.	SMI BeGaze eye-movement analysis software; SteamVR framework.	Eye-tracking sampling rate: 250 Hz; omnidirectional 4K stimulus footage. Exact motion-to-photon latency, tracking accuracy, and detailed headset tracking

						configuration not reported.
**[21]**	MiHiepa/Rezzil soccer simulator delivered through an HTC Vive Pro HMD.	HMD positional tracking with additional HTC Tracker 2.0 sensors attached to the lower limbs.	Task-specific lower-limb interaction through instrumented training shoes/shin guards; additional handheld controller use not confirmed from the available report.	Four HTC Tracker 2.0 sensors attached to the shoes and shin guards.	MiHiepa/Rezzil VR platform.	HMD resolution: 2880 × 1600 pixels; refresh rate: 90 Hz; horizontal and vertical field of view: 110°.
**[13]**	Indoor VR Sports Room used for soccer-related physical-education activities	Camera-based object recognition and movement detection	Body movements and kicking actions; no handheld controller reported	Object-recognition camera system; exact camera model not reported	Real-time image-processing and feedback software; name and version not reported	Camera model, sampling rate, latency, frame rate, field of view, calibration, and recognition accuracy not reported
**[22]**	Helix-Arena 360° immersive projection environment presenting a multiple-object-tracking task	Participant positional-tracking configuration not reported	HTC Vive handheld controller used to record responses	No auxiliary physiological sensors reported	Unreal Engine-based virtual environment reported; additional analysis software not reported	Projection resolution, frame rate, field of view, latency, controller sampling rate, calibration, and tracking accuracy not reported
**[25]**	Immersive first-person HMD-based soccer decision-training environment; HMD model not reported	HMD-based positional tracking; exact architecture not reported	Response method or controller not reported	Eye-tracking or gaze-recording system used for visual-search outcomes; exact hardware model not reported	Gaze-analysis and statistical software not reported	HMD resolution, degrees of freedom, eye-tracker sampling rate, calibration, latency, frame rate, field of view, and tracking accuracy not reported
**[23]**	Helix-Arena 360° immersive projection	Participant positional-tracking	Handheld response controller; exact model not reported	No auxiliary physiological sensors reported	Virtual-environment and analysis software not reported	Projection resolution, latency, frame rate, field of

	environment used for perceptual-cognitive training	configuration not reported				view, controller sampling rate, calibration, and tracking accuracy not reported
**[4]**	CortexVR delivered through an Oculus Rift HMD; non-VR desktop mode used as the within-subject comparator	Oculus Rift positional tracking; exact Rift model and camera configuration not reported	Oculus Touch handheld controllers	No additional physiological sensors reported	CortexVR platform with adaptive and machine-learning-supported task analysis	Motion-to-photon latency 34.4 ms (SD 27.4 ms); frame rate 78.39 frames/s; resolution, field of view, calibration, and tracking accuracy not reported
**[24]**	HMD-based viewing of immersive 360° soccer videos for decision-making assessment	Head-orientation tracking through the HMD; exact headset and positional-tracking configuration not reported	Response device not reported	No auxiliary sensors reported	Decision-test and statistical-analysis software not reported	HMD model, display resolution, degrees of freedom, latency, frame rate, field of view, calibration, and tracking accuracy not reported
**[15]**	Rezzil Player 22 delivered through an Oculus Quest 2 HMD	Camera-based inside-out 6DoF head and controller tracking using the headset's four-camera array	Oculus Quest 2 handheld controllers	No additional physiological sensors reported	Rezzil Player 22 application; video coding used for heading outcomes	Four-camera inside-out tracking reported; motion-to-photon latency, frame rate, display resolution, field of view, calibration, and tracking accuracy not reported
**[16]**	VR balance or exergame rehabilitation environment; exact HMD, display, or	Positional-tracking method not reported	Body-movement and balance responses; controller or platform configuration not reported	EMG and balance-assessment equipment; exact device models not reported	VR rehabilitation and EMG-analysis software not reported	Display specifications, degrees of freedom, EMG sampling rate, calibration, latency, frame rate, and

	projection system not reported					tracking accuracy not reported
**[26]**	Immersive VR soccer scenarios for scanning, anticipation, and pass-selection training; exact display hardware not reported	Positional or head-orientation tracking not sufficiently described	Response or interaction device not reported	No auxiliary physiological sensors reported	VR simulation and behavioral-coding software not reported	Display resolution, tracking architecture, degrees of freedom, latency, frame rate, field of view, sampling rate, calibration, and tracking accuracy not reported
**[5]**	Rezzil-based immersive training delivered through a Valve Index HMD	HMD-based positional tracking; exact base-station configuration not reported	Valve Index controllers and foot-tracking peripherals	EEG electrodes or headset and foot-tracking devices	Rezzil training platform; EEG-processing software not reported	EEG sampling rate, electrode configuration, tracking calibration, latency, frame rate, field of view, and foot-tracker accuracy not reported
**[27]**	SensiballVR immersive soccer-training environment; exact HMD model not reported	HMD-based positional tracking; exact inside-out or external tracking configuration not reported	Handheld controller or task-specific response method not reported	No auxiliary physiological sensors reported	SensiballVR platform; additional analysis software not reported	HMD model, degrees of freedom, latency, frame rate, field of view, calibration, tracking accuracy, and sensor sampling rate not reported
**[28]**	Immersive VR heading-training environment; exact HMD or display model not reported	Positional-tracking architecture not reported	VR heading-response interface; controller or tracked-object configuration not reported	No auxiliary physiological sensors reported	VR heading-training software not reported	Display resolution, degrees of freedom, tracking architecture, latency, frame rate, field of view, calibration, and tracking accuracy not reported

**[29]**	CAVE-type or large-scale immersive projection environment presenting virtual soccer scenes	Participant or viewpoint tracking used within the virtual environment; exact hardware configuration not reported	Task-response device not reported	No auxiliary physiological sensors reported	Virtual soccer-environment and multiple-player-tracking analysis software not reported	Number of projection surfaces, stereoscopic configuration, motion-capture system, sampling rate, latency, frame rate, field of view, calibration, and tracking accuracy not reported
**[30]**	Smartphone-based VR imagery environment used for football-kicking practice	No full-body or room-scale positional tracking reported; smartphone orientation sensing used where applicable	Smartphone or smartphone-based headset viewing; no separate handheld controller reported	No auxiliary physiological sensors reported	VR imagery application; conventional imagery script used as a comparison condition	Smartphone model, headset model, display resolution, frame rate, field of view, latency, orientation-sensor specifications, and calibration not reported
[31]	Immersive VR-based multimodal training system; exact HMD or display model not reported	Not reported	Not reported	Balance and agility assessment instruments; exact device models not reported	Not reported	HMD model, degrees of freedom, latency, frame rate, field of view, sensor sampling rate, calibration, and tracking accuracy not reported
[32]	3-D virtual reality system integrated with small-sided-game training; exact HMD or display model not reported	Not reported	Not reported	Self-report motivation/teamwork scales and technical-performance ratings; no physiological sensors reported	Not reported	HMD model, degrees of freedom, latency, frame rate, field of view, calibration, and tracking accuracy not reported
[33]	VR-assisted coaching environment; exact HMD or display model not reported	Not reported	Not reported	Physical-performance testing equipment (Illinois Agility Test, Yo-Yo IR1, 30-m sprint) and psychological questionnaires (Sport Confidence Inventory, Group Environment	Not reported	HMD model, degrees of freedom, latency, frame rate, field of view, calibration, and tracking accuracy not reported

				Questionnaire, PSS-10); no physiological sensors reported		

Note: Technical classifications were based solely on information explicitly reported in the original studies. Commercial device names were not used to infer unreported tracking architectures. "Not reported" indicates that the source article did not provide sufficient technical information. Helix-Arena was classified as a 360° immersive projection environment rather than as a tracking system. Handheld controllers were classified as interaction or response devices unless their role in positional tracking was explicitly described. SMI BeGaze was classified as eye-movement analysis software rather than as physical eye-tracking hardware. HMD, head-mounted display; IMU, inertial measurement unit; EEG, electroencephalography; EMG, electromyography; 6DoF, six degrees of freedom.
